# Unveiling the Miracle Tree: Therapeutic Potential of *Moringa oleifera* in Chronic Disease Management and Beyond

**DOI:** 10.3390/biomedicines13030634

**Published:** 2025-03-05

**Authors:** Edgar Yebran Villegas-Vazquez, Rocio Gómez-Cansino, Gabriel Marcelino-Pérez, Domingo Jiménez-López, Laura Itzel Quintas-Granados

**Affiliations:** 1 Laboratorio de Farmacogenética, UMIEZ, Facultad de Estudios Superiores Zaragoza, Universidad Nacional Autónoma de México, Batalla 5 de Mayo s/n Esquina Fuerte de Loreto, Ciudad de México 09230, Mexico; eyebran.villegas@gmail.com; 2 Colegio de Ciencias y Humanidades, Plantel Casa Libertad, Universidad Autónoma de la Ciudad de México, Calzada Ermita Iztapalapa 4163, Colonia Lomas de Zaragoza, Ciudad de México 09620, Mexico; rocio.gomez.cansino@uacm.edu.mx; 3 Departamento de Bioquímica y Biología Estructural, Instituto de Fisiología Celular, Universidad Nacional Autónoma de México, Ciudad de México 04510, Mexico; 4 Departamento de Nutrición, Universidad Global Latinoamericana, Av. Vía Adolfo López Mateos 73, Misiones, Naucalpan de Juárez, Méx., Mexico 53250, Mexico; 5 Departamento de Investigación y Desarrollo, Soluciones Orgánicas, Fertilizantes y Servicios para el Agro (SOFESA), Av. Revolución, No. 1267, Ciudad de México 01040, Mexico; 6 Colegio de Ciencias y Humanidades, Plantel Cuautepec, Universidad Autónoma de la Ciudad de México, Av. La Corona 320, Colonia La Palma, Ciudad de México 00000, Mexico

**Keywords:** *Moringa oleifera*, bioactive properties, chronic diseases, phytochemical extraction optimization

## Abstract

*Moringa oleifera* (MO) has gained recognition as a potent natural intervention for preventing and managing chronic diseases (CDs) due to its diverse phytochemical composition and pharmacological properties. Rich in antioxidants, polyphenols, flavonoids, and glucosinolates, MO exerts anti-inflammatory, anti-hyperglycemic, cardioprotective, and anti-obesity effects. These properties make it a valuable therapeutic agent for CDs, including diabetes, cardiovascular diseases, obesity, neurodegenerative disorders, and cancer. MO’s ability to modulate oxidative stress and inflammation—key drivers of CDs—highlights its significant role in disease prevention and treatment. MO enhances insulin sensitivity, regulates lipid profiles and blood pressure, reduces inflammation, and protects against oxidative damage. MO also modulates key signaling pathways involved in cancer and liver disease prevention. Studies suggest that MO extracts possess anticancer activity by modulating apoptosis, inhibiting tumor cell proliferation, and interacting with key signaling pathways, including YAP/TAZ, Nrf2-Keap1, TLR4/NF-κB, and Wnt/β-catenin. However, challenges such as variability in bioactive compounds, taste acceptability, and inconsistent clinical outcomes limit their widespread application. While preclinical studies support its efficacy, large-scale clinical trials, standardized formulations, and advanced delivery methods are needed to optimize its therapeutic potential. MO’s multifunctional applications make it a promising and sustainable solution for combating chronic diseases, especially in resource-limited settings.

## 1. Introduction

Noncommunicable diseases, or chronic diseases (CDs), refer to long-lasting conditions or illnesses that persist for at least three months and may lead to serious long-term effects. While CDs are more prevalent among older adults, they are typically manageable but not curable [1]. Common types of CDs include cancer, cardiovascular disease (CVD), heart disease, diabetes, neurological disorders, liver disease, stroke, and arthritis [2].

In 2021, CDs were responsible for at least 43 million deaths, accounting for 75% of global non-pandemic-related fatalities. Of these, 18 million occurred in individuals under 70, with 82% of premature deaths concentrated in low- and middle-income countries. CVDs were the leading cause, contributing to at least 19 million deaths, followed by cancer (10 million), chronic respiratory diseases (4 million), and diabetes (over 2 million, including kidney disease linked to diabetes). These four disease groups account for 80% of premature CD deaths [3].

By 2030, the global cost of CDs is projected to reach USD 47 trillion. Individual lifestyle choices, behaviors, and community factors are crucial in developing and managing these conditions. Many CDs, including diabetes, heart disease, and respiratory illnesses, are largely preventable, with key risk factors such as physical inactivity, unhealthy diet, tobacco use, and excessive alcohol consumption. However, investment in prevention remains disproportionately low compared to treatment, both in terms of lifestyle changes and addressing social determinants of health [4].

Given the rising burden of CDs, advancements in technology and pharmaceuticals, alongside increased investment in prevention, will be essential for a sustainable future. In this context, natural bioactive compounds present a promising alternative for treating and preventing CDs. In this review, we focus on the beneficial properties of *Moringa oleifera* (MO), highlighting its significance in combating CDs. We provide comprehensive insights into its phytochemical composition and pharmacological properties, including antioxidant, anticancer, hepatoprotective, antimicrobial, antiviral, anti-inflammatory, and neuroprotective effects. Collectively, these properties make MO a promising candidate for preventing and managing various CDs.

## 2. *Moringa oleifera*

*M. oleifera*, known as the “miracle tree”, is native to India, Afghanistan, Bangladesh, and Pakistan but thrives in tropical and subtropical regions worldwide. It has spread to Asia, Africa, the Caribbean, Latin America, the Pacific Islands, and beyond, and it was introduced from India to regions like Africa and the Philippines in ancient times. The tree grows best in warm temperatures (25–35 °C), indirect sunlight, and slightly acidic to alkaline soil without waterlogging. It begins bearing fruit within 6 to 8 months and is commercially cultivated in areas such as Africa, Mexico, Hawaii, and South America, although its nutrient content varies depending on soil conditions [5]. The taxonomic classification of MO is shown in Table 1.

Furthermore, the Moringaceae family comprises the species *M. arborea*, *M. borziana*, *M. concanensis*, *M. drouhardii*, *M. hildebrandtii*, *M. longituba*, *M. oleifera*, *M. ovalifolia*, *M. peregrina*, *M. pygmaea*, *M. rivae*, *M. ruspoliana*, and *M. stenopetala* [6].

*Moringa oleifera* extracts from its leaves and seeds contain phytoconstituents that offer health benefits, including antibacterial, antifungal, anti-inflammatory, and antioxidant properties. They also support heart and liver health, assist in fertility treatments, and promote wound healing in diabetic patients [5]. The Moringaceae family has a rich history in traditional medicine, referenced in ancient texts along the Silk Road and esteemed in Ayurveda. Moringa species hold cultural, spiritual, and religious significance in regions such as the Arabian Peninsula [7,8].

## 3. Phytochemistry of Moringa

Moringa contains various phytochemicals, and their levels depend on several environmental factors, extraction methods, and the plant parts used, among others. These compounds have pharmacological benefits that may assist in managing CDs. Previous reports suggest that methanolic, ethanolic, or aqueous extracts from leaves or seeds of the Moringa genus include carotenoids, tocopherols, phenolic acids and phenols, folates, glucosinolates, alkaloid, tannins, flavonoids, saponins, terpenoids, sterols, esters, glycosides, carbamates, isothiocyanates, steroids, coumarins, fatty acids, polyunsaturated fatty acids, polysaccharides, mineral salts, vitamins, proteins, and amino acids, among others [5,9,10,11,12,13]. A proximal analysis of Moringa chemical composition is shown in Table 2.

Over 330 compounds have been identified in *Moringa oleifera* using analytical methods such as HPLC and GC-MS, including phenols, flavonoids, glucosinolates, alkaloids, vitamins, minerals, and fatty acids (Table 3). However, fewer than a third have been quantified. *M. oleifera* seeds contain 43.56% oil, primarily composed of unsaturated fatty acids, with oleic acid as the major component (74.99%), followed by palmitic acid (12.51%), lauric acid (1.97%), stearic acid (2.09%), linoleic acid (1.27%), and linolenic acid (1.75%). Additionally, the seeds contain 145.16 mg/100 g of total polyphenols, 144.07 mg/kg of flavonoids, 140.49 mg/kg of proanthocyanidins, 40.17 mg GAE (gallic acid equivalents)/g of total phenols, and 18.24 mg RE/g of total flavonoids [32,33].

The seeds and leaves of *M. oleifera* are rich in flavonoids such as myricetin (5.8 mg/g), rutin (555.6 µg/g), kaempferol (197.6 µg/g), quercetin (2030.9 µmol/100 g), and isorhamnetin (0.118 mg/g) and phenols such as gallic acid (1.034 mg/g), caffeic acid (0.409 mg/g), O-coumaric acid (0.536 mg/g), and ellagic acid (0.078–0.128 mg/g) [31]. In addition, Anwar and Rashid reported that the most abundant sterols in the oil of moringa seeds were β-sitosterol (46.16%), campesterol (17.59%), stigmasterol (18.80%), and ∆^5^-avenasterol (9.26%) [34]; these results are consistent with those reported by Lalas and Tsaknis [35] but differ from those reported by Abd El Baky and El-Baroty [36] probably as a consequence of the environmental factors, genetic character, fertilization, and growth conditions of moringa plants.

**Table 3:** Phytochemical composition of *M. oleifera*.

Compound	Plant Part	Ref
Phenols and Phenolic Acids		
1-*O*-phenyl-α-L-rhamnopyranoside	Seeds	[37]
2-Hydroxylcoumaric acid	Leaves	[38]
3,4-dihydroxybenzoic acid	Leaves	[39]
3-caffeoylquinic acid	Leaves	[40]
4-(α-l-rhamnopyranosyloxy) Benzaldehyde	Root barks	[41]
4-(α-l-rhamnosyloxy) phenylacetonitrile (niazirin)	Seeds	[39]
4-Aminobenzoic acid	Root barks	[41]
4-caffeoylquinic acid	Leaves	[42,43]
4-*O*-(3′-*O*-α-D-glucopyranosyl)-caffeoyl quinic acid	Leaves	[39,44]
4-*O*-(4′-*O*-α-D-glucopyranosyl)-caffeoylquinic acid	Leaves	[39,44]
4-*O*-caffeoylquinic acid	Leaves	[39,44]
4-*O*-β-D-glucopyranoside benzoic acid	Leaves	[39,42]
5-caffeoylquinic acid	Leaves	[40]
5-*O*-caffeoylquinic acid	Leaves	[39,44]
7-*O*-(β-d-glucopyranosyl)-5-*O*-Menaringenin-4′-[α-l-rhamnopyranosyl-(1→2)]-β-d-glucopyranoside	Leaves	[45]
Benzaldehyde 4-*O*-α-L-rhamnopyranoside	Leaves	[42]
Benzaldehyde-4-*O*-β-glucoside	Leaves	[46]
Benzoic acid	Stems	[41]
Benzoic acid 4-*O*-α-rhamnosyl-(1→2)-β-glucoside	Leaves	[46]
Benzoic acid-4-*O*-β-glucoside	Leaves	[46]
Benzyl β-d-arabinopyranosyl-(1→6)-β-d-glucopyranoside	Stems	[41]
Benzyl β-d-xylopyranosyl-(1→6)-β-d-glucopyranoside	Leaves	[47]
Benzyl-β-d-glucopyranoside	Leaves	[47]
Caffeic acid	Leaves, seeds	[48,49]
Caffeoylquinic acid	Leaves	[39,42]
Chlorogenic acid	Leaves	[39,44]
Cinnamic acid	Seeds	[49]
Cryptochlorogenic acid	Leaves	[39,50]
Ellagic acid	Leaves, seeds	[48,51]
Epicatechin	Leaves	[52]
Ferulic acid	Leaves, seeds	[48,49]
Gallic acid	Leaves, seeds	[48,51,53]
Gentistic acid	Leaves	[38]
Methyl 4-caffeoylquinate	Leaves	[39]
Methyl caffeoylquinate	Leaves	[39,42]
*O* coumaric acid	Leaves	[38,48]
*p*-Coumaric acid	Seeds	[49]
Pirocatechin	Leaves	[29,54,55,56]
*p*-Hydroxybenzaldehyde	Stems, root barks	[41]
*p*-Hydroxybenzoic acid	Stems	[41]
Protocatechuic acid	Seeds	[49]
Pyrogallol	Leaves	[54,55,56]
Sinapic acid	Leaves	[38,48]
Syringic acid	Leaves	[38,48,57]
*Trans*-ferulic acid	Stems	[41]
Vanillin	Seeds	[39,58]
α-Tocopherol	Leaves	[59]
γ-Tocopherol	Leaves	[59]
Flavonoids		
3,5,6-trihydroxy-2-(2,3,4,5,6-pentahydroxyphenyl)-4*H*chrome*n*-4-one	Leaves	[39]
4-Hydroxymellein	Stems	[58]
Apigenin	Leaves	[56]
Apigenin-*O*-8-glucoside	Leaves	[60]
Astragalin	Flowers, leaves	[39,43]
Catechin	Seeds	[49]
Daidzein	Leaves	[52]
Genistein	Leaves	[52]
Isoquercitrin	Flowers, leaves	[43,61]
Isorhamnetin	Leaves	[48]
Isorhamnetin 3-*O*-(6″-malonylglucoside)	Pods	[62]
Kaempferide-3-*O*-(2″-*O*-galloylrhamnoside)	Leaves	[46]
Kaempferide-3-*O*-(2″-*O*-galloylrutinoside)-7-*O*-α-rhamnoside	Leaves	[46]
Kaempferide-3-*O*-2″,3″-diacetylglucoside	Leaves	[46]
Kaempferitrin	Flowers	[61]
Kaempferol	Leaves	[51,63]
Kaempferol acetyl glycoside	Leaves	[39]
Kaempferol-3-*O*-β-D-(6″-*O*-malonyl)-glucoside	Leaves	[47]
Kaempferol-3-*O*-(6″-malonyl-glucoside)	Leaves	[39,40]
Kaempferol-3-*O*-[α-rhamnosyl-(1→2)]-[α-rhamnosyl-(1→4)-β-glucoside-7-*O*-α-rhamnoside	Leaves	[46]
Kaempferol-3-*O*-[β-glucosyl-(1→2)]-[α-rhamnosyl-(1→6)-*O*-β-glucoside-7-*O*-arhamnoside	Leaves	[46]
Kaempferol-3-*O*-β-d-glucopyranoside	Leaves	[64]
Kaempferol-3-*O*-[methyl-(S)-3-hydroxy-3-methylglutaroyl (1→6)]-β-d-glucopyranoside	Leaves	[65]
Kaempferol-3-*O*-glucoside	Leaves	[40]
Kaempferol-*O*-7-glucoside	Leaves	[60]
Kaempferol-3-*O*-rutinoside	Pods	[43,62]
Kaempferol-*O*-3,7-diglucoside	Leaves	[60]
Kaempferol-malonyl-glycoside	Leaves	[43]
Luteolin	Leaves	[56]
Myricetin	Seeds and leaves	[48]
Procyaniadin	Seeds	[66]
Quercetin	Leaves	[67]
Quercetin-3-acetylglucoside	Leaves	[60]
Quercetin 3-*O*-rhamnoglucoside (rutin)	Leaves	[39,40,43,68,69]
Quercetin 3-*O*-β-D-glucopyranoside	Leaves	[42]
Quercetin-3-*O*-(6″-malonyl-glucoside)	Leaves	[39,40]
Quercetin-3-*O*-6-[(E)-4-methoxy-5-methylhexa-2, 4-dienoatyl (1→ 6)]-β-d-glucopyranoside	Leaves	[65]
Quercetin-3-*O*-6-[methyl-(S)-3-hydroxy-3-methylglutaroyl (1→ 6]-β-d-glucopyranoside	Leaves	[65]
Quercetin-*O*-3,7-diglucoside	Leaves	[60]
Quercetin 3-*O*-rutinoside	Pods	[62]
Quercetin-3-*O*-glucoside	Leaves, seeds	[40,62]
Quercetin-3-*O*-β-D-(6″-*O*-3-hydroxy-3-methylglutaryl)-glucoside	Leaves	[39]
Quercetin-acetyl-glycoside	Leaves	[43]
Vitexin	Leaves	[39,43]
Glucosinolate		
4-[(2′-*O*-acetyl-α-l-rhamnosyloxy) benzyl] isothiocyanate	Fruits	[70]
4-[(3′-*O*-acetyl-α-l-rhamnosyloxy) benzyl] isothiocyanate	Fruits	[70]
3-hydroxy-4-(α-l-rhamnosyloxy)-benzyl glucosinolates	Seeds	[71]
4-(2′-acetyl-α-l-rhamnosyloxy)-benzyl glucosinolates	Seeds	[71]
4-(3′-acetyl-α-l-rhamnosyloxy)-benzyl glucosinolates	Seeds	[71]
4-(3′-*O*-acetyl-α-L-rhamnosyloxy) benzyl isothiocyanate	Seeds	[39]
4-(4′-acetyl-α-l-rhamnosyloxy)-benzyl glucosinolates	Seeds	[71]
4-(α-L-rhamnopyranosyloxy)-benzylglucosinolate	Seeds, leaves	[39]
4-*O*-(α-L-rhamnopyranosyloxy)-benzyl glucosinolate (glucomoringin)	Leaves, barks, seeds	[40,72]
4-(α-L-rhamnosyl) benzyl ethyl ester	Leaves, seeds, flowers	[39]
4-[(2′-*O*-acetyl-α-l-rhamnosyloxy)benzyl]isothiocyanate	Pods	[70]
4-[(3′-*O*-acetyl-α-l-rhamnosyloxy)benzyl]isothiocyanate	Pods	[70]
4-[(4′-*O*-acetyl-α-l-rhamnosyloxy)benzyl]isothiocyanate	Pods, leaves	[40,70]
4-[(α-l-rhamnosyloxy)benzyl]isothiocyanate	Pods	[70]
4′-*O*-acetyl-4-(α-l-rhamnosyloxy)-benzyl glucosinolates	Seeds	[73]
4-*O*-(α-l-acetylrhamnopyranosyloxy)-benzyl glucosinolate isomer 1	Roots	[40]
4-*O*-(α-l-acetylrhamnopyranosyloxy)-benzyl glucosinolate isomer 2	Roots	[40]
4-*O*-(α-l-acetylrhamnopyranosyloxy)-benzyl glucosinolate isomer 3	Roots	[40]
Benzylglucosinolate	Seeds, roots	[40,61,71]
Glucobarbarin	Seeds	[71]
Glucoiberin	Seeds	[71]
Glucomoringin	Seeds	[63,71,74]
Glucoraphenin	Seeds	[71]
Glucosinalbin	Seeds	[71]
Glucotropaedlin	Seeds	[71]
Moringaside C	Leaves	[39]
Moringaside D	Leaves	[39]
Moringaside E	Leaves	[39]
Moringaside F	Leaves	[39]
Moringaside G	Leaves	[39]
Moringin	Seeds	[39]
Niazirin	Seeds, leaves	[70]
Niazirinin	Leaves	[75]
Carbamates		
4-[(β-d-glucopyranosyl)-(1→3)-(α-l-rhamnopyranosyl)] phenylacetonitrile	Fruits	[37]
4-(β-d-glucopyranosyl-1→4-α-l-rhamnopyranosyl)benzyl thiocarboxamide	Seeds	[76]
4-[(2′,3′,4′-tri-*O*-acetyl-α-l-rhamnosyloxy)benzyl] nitrile	Leaves	[77]
Me-*N*-4-(α-l-rhamnopyranosyloxy)-benzyl carbamate	Seeds	[76]
Methyl-4-(α-l-rhamnopyranosyloxy)benzyl carbamate	Leaves	[39,47]
Methyl-*N*-(4-[(4′-*O*-acetyl-α-l-rhamnopyranosyl)benzyl]) carbamate	Seeds	[37]
*N*-[4-(β-l-rhamnopyranosyl)benzyl]-1-*O*-α-d-glucopyranosyl-thiocarboxamide	Seeds	[37]
*N*-benzylcarbamic acid	Root barks	[41]
Niazicin A	Pods	[75]
Niazidin	Pods	[78]
Niazimicin	Leaves, seeds	[79,80]
Niazimimin A	Leaves	[80]
Niazimimins B	Leaves	[80]
Niazinin	Seeds	[39]
Niazinin A	Pods, Leaves	[70]
Niazinin B	Leaves and seeds	[39,80]
*O*-ethyl,4-[(2′,3′,4′-tri-*O*-acetyl-α-l-rhamnosyloxy)benzyl] thiocarbamate (Z)	Leaves	[77]
*O*-ethyl-4-[(2′,3′,4′-tri-*O*-acetyl-α-L-rhamnosyloxy) benzyl] carbamate (E)	Leaves	[77]
*O*-ethyl-4-[(2′,3′,4′-tri-*O*-acetyl-α-L-rhamnosyloxy) benzyl] thiocarbamate (E)	Leaves	[77]
*O*-ethyl-4-[(α-l-rhamnopyranosyloxy)-3-hydroxybenzyl]thiocarbamate	Seeds	[75]
*O*-ethyl-4-[(α-L-rhamnosyloxy)-benzyl] carbamate	Pods	[81]
*O*-ethyl-4-[α-l-rhamnosyloxy)benzyl] thiocarbamate(Z)	Leaves	[77]
*O*-ethyl-p-hydroxy benzyl thiocarbamate	Pods	[78]
*O*-methyl-4-[(2′,3′,4′-tri-*O*-acetyl-α-L-rhamnosyloxy) benzyl] carbamate (E)	Leaves	[77]
*O*-methyl-4-[(2′,3′,4′-tri-*O*-acetyl-α-L-rhamnosyloxy) benzyl] carbamate (Z)	Leaves	[77]
*O*-methyl-4-[(2′,3′,4′-tri-*O*-acetyl-α-L-rhamnosyloxy) benzyl] thiocarbamate (E)	Leaves	[77]
*O*-methyl-4-[(2′,3′,4′-tri-*O*-acetyl-α-L-rhamnosyloxy)benzyl] thiocarbamate (Z)	Leaves	[77]
*O*-methyl-4-[(4′-*O*-acetyl-α-L-rhamnosyloxy) benzyl] carbamate (E)	Leaves	[77]
*O*-n-butyl-4-[(α-l-rhamnopyranosyloxy) benzyl]thiocarbamate	Seeds	[75]
S-methyl-N-(4-[(α-l-rhamnopyranosyloxy)benzyl])thiocarbamate	Leaves	[70]
S-methyl-N-(4-((α-l-rhamnosyloxy) benzyl)) thiocarbamate	Fruits	[70]
S-methyl-N-thiocarbamate	Pods	[75]
Isothiocyanates		
4-(4′-*O*-acetyl-α-L-rhamnosyloxy) benzyl isothiocyanate	Seeds	[79]
4-(α-L-rhamnosyloxy)benzyl isothiocyanate	Seeds	[79]
4-[(3′-*O*-acetyl-α-L-rhamnosyloxy) benzyl] isothiocyanate	Seeds	[79]
4-(α-l-rhamnosyloxy) benzyl isothiocyanate	Seeds	[61,82]
4 (α-L-rhamnosyloxy) isothiocyanate	Seeds	[83]
Pterygospermin	Root barks, leaves, pods	[84]
Sulforaphane	Pods	[85]
Sterols		
24-methylene-9,19-cyclolanostan-3-ol	Flowers	[86]
24-methylene-cholesterol	Seeds	[87]
28-isoavenasterol	Seeds	[87]
3-*O*-(6′-*O*-oleoyl-β-d-glucopyranosyl)-β-sitosterol	Seeds	[88]
Campestanol	Seeds	[87]
Campesterol	Seeds	[87]
Clerosterol	Seeds	[87]
Stigmastanol	Seeds	[87]
Stigmasterol	Seeds, pods	[63,79,87]
β-sitosterone	Flowers, roots	[59,86]
β-sitosterol	Seeds, stems	[61,79,87,89,90]
β-sitosterol-3-*O*-glucoside	Seeds, pods	[63]
β-Sitosterol-3-*O*-β-d-glucopyranoside	Seeds	[90]
β-sitosteryl oleate	Seeds	[5,79]
Δ5-avenasterol	Seeds	[87]
Δ7-avenasterol	Seeds	[87]
Δ7.14-stigmastanol	Seeds	[87]
Δ7-campestanol	Seeds	[87]
Fatty acids		
3,4-methylene azelaic acid	Leaves, seeds, flowers	[39]
Behanic acid	Leaves, seeds	[87,91]
Caprylic acid	Leaves	[91]
Eicosenoic acid (arachidic acid)	Seeds	[87,92,93]
Erucic acid	Seeds	[87,92]
Glycerol-1-(9-octadecanoate)	Seeds	[39]
Heicosanoic acid	Leaves	[91]
Heneicosanoic acid	Leaves, seeds, flowers	[39]
Lauric acid	Leaves	[91]
Ligoceric acid	Leaves	[91]
Linolenic acid	Leaves	[91]
Margaric acid	Leaves	[91]
Monacosan-15-one	Leaves, seeds, flowers	[39]
Monoacetyl glycerol	Leaves, seeds, flowers	[39]
Myristic acid	Seeds	[91,94]
Octacosanoic acid	Stems	[95]
Oleic acid	Leaves, seeds	[63,79,87]
Palmitic acid	Leaves, seed	[91,92,96]
Stearic acid	Leaves, seeds	[87,91,92]
Tricosanoic acid	Leaves	[91]
Triolein	Seeds	[79]
Triolein acid	Leaves, seeds, flowers	[39]
Vaccenic acid	Leaves	[91]
α-linolenic acid	Leaves, seeds	[91]
α-tocopherol	Seeds	[96]
β-tocopherol	Seeds	[96]
γ-linolenic acid	Leaves	[91]
δ-tocopherol	Seeds	[96]
Esters		
Methyl-4-(α-L-rhamnopyranosyloxy) benzyl carbamate	Leaves, flowers, seeds	[39]
*O*-ethyl-4-[(α-l-rhamnosyloxy)-benzyl] carbamate	Whole pods	[61]
Alkaloids		
4-(α-L-rhamnopyranosyloxy)phenylacetonitrile (niazirin)	Leaves	[72]
4′-hydroxyphenylethanamide (marumosides A and B)	Leaves	[47]
4″-*O*-α-L-rhamnopyranoside, methyl 4-(α-L-rhamnopyranosyloxy)-benzylcarbamate	Leaves	[72]
4-hydroxymellein	Stems	[61]
5-dehydroxymethyl-pyrrolemarumine 4″-*O*-α-l-rhamnopyranoside	Leaves	[65]
Aurantiamide acetate	Leaves	[39]
Hostine D	Seeds	[39]
Marumoside A	Leaves, roots	[39,47]
Marumoside B	Leaves, roots	[39,47]
Moringin	Stems	[97]
Moringinine	Stems	[98]
*N*1-methyl-N2-((4-*O*-α-l-rhamnopyranoside)benzyl) oxalamide	Leaves	[65]
*N*,α-L-rhamnopyranosylvincosidamide	Leaves	[72]
Pyrrolemarumine	Leaves	[39]
Pyrrolemarumine 4″-*O*-α-l-rhamnopyranoside	Leaves	[47]
Pyrrolemorine A	Leaves	[39]
Pyrrolemorine B	Leaves	[39]
Pyrrolemorine C	Leaves	[39]
Pyrrolemorine D	Leaves	[39]
Pyrrolemorine E	Leaves	[39]
Pyrrolemorine F	Leaves	[39]
Pyrrolemorine G	Leaves	[39]
Tangutorid E	Seeds	[39]
Tangutorid F	Seeds	[39]
Carotenoids		
13-*cis*-lutein	Leaves	[26,29,38,99]
15-*cis*-β-carotene	Leaves	[26,29,38,99]
Lutein	Leaves	[100]
*trans*-zeaxanthin	Leaves	[26,29,38,99]
*trans*-lutein	Leaves	[26,29,38,99]
*trans*-luteoxin	Leaves	[26,29,38,99]
β-Carotene	Leaves	[100]
Vitamins		
Vitamin B1, vitamin B2, vitamin B3, vitamin C, vitamin E, β-carotene, α-tocopherol	Leaves	[26,29,38,72,99]
Vitamin C	Flowers, pods	[101]
α-Tocopherol (vitamin A), γ-tocopherol, δ-tocopherol	Seeds	[87]
Minerals		
Magnesium, potassium, calcium, iron	Roots, barks	[102]
Zinc, phosphorus, magnesium, manganese, copper, potassium, selenium, sulfur, sodium, calcium, iron	Leaves, seeds, pods	[34,102]
Amino acids		
Alanine	Leaves, pods, seeds	[29,56,91]
Arginine	Leaves, pods, seeds	[29,56,91]
Aspartic acid	Leaves, pods, seeds	[29,56,91]
Cysteine	Leaves, pods, seeds	[29,56,91]
Glutamic acid	Leaves, pods, seeds	[29,56,91]
Glycine	Leaves, pods, seeds	[29,56,91]
Histidine	Leaves, pods, seeds	[29,56,91]
Hydroxyproline	Leaves, seeds	[29,56,91]
Isoleucine	Leaves, pods, seeds	[29,56,91]
Leucine	Leaves, pods, seeds	[29,56,91]
Lysine	Leaves, pods, seeds	[29,56,91]
Methionine	Leaves, pods, seeds	[29,56,91]
Phenylalanine	Leaves, pods, seeds	[29,56,91]
Proline	Leaves, pods, seeds	[29,56,91]
Serine	Leaves, pods, seeds	[29,56,91]
Threonine	Leaves, pods, seeds	[29,56,91]
Tretinoin	Leaves, seeds	[29,56,91]
Tryptophan	Leaves, pods, seeds	[29,56,91]
Tyrosine	Leaves, pods, seeds	[29,56,91]
Valine	Leaves, pods, seeds	[29,56,91]
Others		
1-octadecene	Seeds	[79]
1,3-Dibenzyl urea	Roots	[59]
2-formyl-5-methyl-1*H*-pyrrol-1-ylbutanoic acid	Leaves, flowers, seeds	[39]
2,3,4-trihydroxybenzaldehyde	Seeds, pods	[63]
3,4-dihydroxy-benzoic acid	Leaves	[42]
3,5,6-trihydroxy-2-(2,3,4,5,6-pentahydroxyphenyl)-4*H*-chromen-4-one	Seeds, pods	[63]
3,7,11,15-tetramethyl-2-headecen-1-ol	Flowers	[86]
3-pyridinecarboxamide	Leaves	[42]
4-(α-l-rhamnopyranosyloxy)-benzylglucosinolate carbamate	Seeds	[61]
5-hydroxymethyl-2-furaldehyde	Leaves	[42]
5-hydroxymethyl-2-furancarboxylic acid	Leaves	[42]
Aglycon of deoxy-niazimicine (*N*-benzyl, S-ethylthioformate)	Root barks	[103]
Aurantiamide acetate	Root barks	[41]
Cysteine	Seeds	[100]
De-*O*-methyllasiodiplodin	Root barks	[41]
D-mannose	Flowers	[104]
Ethyl geranyl acetate	Leaves, flowers, seeds	[39,86]
Ethyl-(*E*)–undec-6-enoate	Leaves, flowers, seeds	[39,63]
Heneicosanoic acid	Flowers	[86]
Lasiodiplodin	Stems	[41]
Methionine	Seeds	[100]
Methyl-1-amino pentasulfide-5-sulfinate	Pods	[78]
Methyl 2-[4-(α-l-rhamnopyranosyl)phenyl]acetate	Seeds	[37]
Methyl ferulate	Stems	[41]
Methyl heptanoate	Flowers	[39,86]
Methyl-*p*-hydroxybenzoate	Whole pods, root barks	[41,59,61]
Methyl vanillate	Root barks	[41]
Moringyne	Seeds	[105,106]
*N*,*N*′-bis(4-[(α-l-rhamnosyloxy) benzyl]) thiourea	Seeds	[107]
*N*,α-L-rhamnopyranosyl vincosamide	Leaves	[48]
Nitrile	Whole pods	[61]
Nonacosan-15-one	Flowers	[86]
Octacosanol	Leaves, seeds, flowers	[39]
Phenylacetonitrile	Root barks	[41]

Deep eutectic solvents (DESs) have emerged as eco-friendly alternatives to traditional organic solvents for extracting bioactive compounds from *M. oleifera* leaves. The choline chloride (ChCl)/1,4-butanediol combination proved most effective in maximizing total phenolic content (TPC), total flavonoid content (TFC), and antioxidant activity (AA), with pulsed ultrasonic-assisted extraction further enhancing yields, particularly of *p*-coumaric acid. Chemometric analysis confirmed the superiority of this DES system. [108]. Table 4 summarizes the techniques used to enhance the extraction of phytochemical compounds from MO.

## 4. The Connection Between Moringa’s Properties and Its Potential to Alleviate Chronic Diseases

Moringa species contain several bioactive metabolites that may be used for the treatment or prevention of CDs due to their beneficial properties, including hepatoprotective, antioxidant, neuroprotective, antidepressant, anticancer, anti-inflammatory, blood glucose-lowering, wound-healing, antibacterial, and antifungal activities [113,114,115,116,117,118], among others, which are discussed in the following paragraphs.

MO leaves contain bioactive components beneficial in managing CDs, such as hypercholesterolemia, high blood pressure, diabetes, insulin resistance, non-alcoholic liver disease, cancer, and inflammation [119,120]. MO is widely recognized for its potent antioxidant properties, attributed to its high content of bioactive compounds such as flavonoids, phenolic acids, isothiocyanates, tannins, saponins, and vitamins. Flavonoids, phenolic acids, and vitamins help neutralize free radicals and reduce oxidative stress, a key factor in CD development [5,11,101,121]. MO’s compounds inhibit lipid peroxidation and enhance the body’s antioxidant defenses [122].

### 4.1. Nutraceutical Properties

Moringa is widely used in traditional medicine across the globe to treat various ailments, including skin infections, anemia, anxiety, asthma, blood impurities, bronchitis, chest congestion, cholera, infections, fever, glandular swelling, headaches, abnormal blood pressure, hysteria, joint pain, pimples, psoriasis, respiratory disorders, scurvy, semen deficiency, sore throat, sprains, tuberculosis, and intestinal worms and for supporting lactation, diabetes management, and pregnancy. Moringa is a food source in low- and middle-income countries due to its availability and its nutritional profile. MO has been used to address malnutrition, encompassing undernutrition, micronutrient deficiencies, and overnutrition. Given the increasing focus on the consumption of fruits, vegetables, and nuts to combat malnutrition, Moringa’s nutritional profile, rich in polyunsaturated fatty acids (PUFAs) compared to saturated fatty acids (SFAs), makes it a valuable dietary resource and has gained a potential and increasing exploration as a nutraceutical [123,124]. Moringa is a highly nutritious plant, rich in essential minerals, proteins, and vitamins. Its leaves are a significant source of vitamins and minerals.

The vitamin C content in the tender leaves of MO plants ranges from 62.66 to 143.59 mg/100 g, while matured leaves contain 51.23 to 150.16 mg/100 g. The flowers have a vitamin C concentration of 77.50 to 224.67 mg/100 g, whereas four-week-old pods contain significantly lower amounts, ranging from 3.96 to 8.27 mg/100 g [72]. Moringa leaves contain 17.5 mg of iron per 100 g of dry weight [125], leaf powder contains 28.2 mg/100 g, fresh leaves contain 0.85 mg/100 g, and dry leaves contain 25.6 mg of iron/100 g [126]. Fresh MO leaves contain vitamin A with 15 mg of β-carotene per 100 g of fresh weight [127]. The zinc content in MO leaves ranges from 25.5 to 31.03 mg/100 g [128]. Moringa fresh leaves contain 440 mg of calcium per 100 g, leaf powder contains 2003 mg/100 g, and dry leaves contain 2185 mg of calcium/100 g [126].

The potassium content in Moringa fresh leaves is 259 mg/100 g, dry leaves contain 1236 mg/100 g, and leaf powder contains 1324 mg/100 g [126]. The amounts mentioned above suggest that MO could be used as a source of minerals and vitamins in the absence of other sources, such as milk, carrots, yogurt, bananas, and spinach. MO leaves have a high protein content, ranging from 22.99% to 29.36% while being low in fat (4.03–9.51%), fiber (6.00–9.60%), and ash (8.05–10.38%) [129].

Immature pods are also nutrient-dense, providing 30% to 46.78% fiber and 2.10% to 20.66% protein. Moringa leaves, pods, and flowers contain 2.2% to 44%, 2.9% to 31%, and 4.79% to 30% amino acids, respectively, including essential amino acids like arginine and histidine, which are crucial for infant nutrition [124,130,131,132,133]. The plant is also a source of PUFAs, such as linolenic and oleic acids, mainly present in immature pods and flowers. Moringa seed oil comprises about 0.89% to 76% PUFAs [124,134]. Moringa oil is characterized by its yellow color and peanut-like flavor, making it a distinctive choice for culinary applications [135]. Its high oleic acid content (70–79%) provides excellent thermal and oxidative stability, making it suitable for frying and cooking [20,135]. The distinctive properties of Moringa oil make it an asset in the food industry. It produces functional foods, such as dressings, that enhance stability and impart desirable sensory characteristics [136]. Furthermore, blending Moringa oil with other oils improves the oxidative stability of the final oil blends, making them ideal for household cooking and deep-frying [137]. However, environmental factors such as location, climate, season, and cultivation conditions can affect its nutritional value. For instance, vitamin A content is higher during the hot–wet season, while vitamin C and iron are higher during the cool–dry season. Additionally, preparation methods and the age of the leaves and pods can influence the nutritional profile of Moringa [124]. The seeds of Moringa possess coagulant and biosorbent properties, enabling their use in water purification and microbial load reduction [138].

### 4.2. Antioxidant and Anti-Inflammatory Properties for Management CDs

Free radicals contribute to the progression of CDs, such as cancer, arthritis, cardiovascular issues, and hypertension, by damaging vital biological components. Polyphenols, as natural plant antioxidants, can neutralize free radicals, protecting health. Moringa seeds demonstrate strong antioxidant properties. Research indicates that defatted Moringa seed flour exhibits higher antioxidant and antibacterial activities, particularly regarding its bound phenolics extract. These extracts demonstrated potent DPPH radical scavenging abilities, suggesting that Moringa seeds could be a valuable source of antioxidants for food, agriculture, and pharmaceuticals [118]. Methanol extraction from bark, pods, stem, and leaves resulted in a mean inhibition of DPPH radicals of 83.62% ± 1.32%, 38.10 ± 1.35%, 66.85 ± 1.20%, and 58.62 ± 1.13%, respectively. Similarly, hexane extraction from bark, pods, stem, and leaves showed a mean inhibition of DPPH radicals of 27.24 ± 1.51%, 15.98 ± 1.24%, 16.05 ± 2.10%, and 32.91 ± 1.63%, respectively [139].

Extracts from MO exhibit strong antioxidant properties through various bioactive compounds, including glycosylates, isothiocyanates, thiocarbamates, flavonoids, kaempferol, myricetin, isoquercetin, astragalin, and crypto-chlorogenic acid [101,140,141,142,143]. The half-maximal inhibitory concentration (IC_50_) values of hydroalcoholic Moringa extract vary depending on the collection time, ranging from 124.51 to 208.81 μg/mL, 516.37 to 900.07 μmol TE/g, 4556.42 to 5959.06 μmol TE/g, and 397.50 to 689.24 μmol TE/g, as determined by DPPH, FRAP (ferric-reducing antioxidant power), ORAC (oxygen radical absorbance capacity), and PCL (photochemiluminescence), respectively. Similarly, methanolic Moringa extract exhibits IC_50_ values ranging from 138.61 to 218.54 μg/mL, 489.37 to 691.09 μmol TE/g, 4215.86 to 4743.70 μmol TE/g, and 416.67 to 640.64 μmol TE/g, as determined by DPPH, FRAP, ORAC, and PCL, respectively [144].

Myricetin isolated from seeds is a more potent antioxidant IC_50_ = 10.5 μM as measured by DPPH than butylated hydroxytoluene (BHT) and alpha-tocopherol [145,146]. Furthermore, MO combined with piperine and curcumin alleviates oxidative stress caused by beryllium toxicity in Wistar rats, suggesting a synergistic effect [143]. Clinical observations suggest that in healthy volunteers, MO reduced plasma malondialdehyde (MDA) and fasting plasma glucose (FPG) levels, demonstrating dose-dependent antioxidant effects without toxicity at doses up to 100 mg/kg [147].

Additionally, aqueous Moringa extract has IC_50_ values ranging from 168.97 to 225.25 μg/mL, 384.56 to 561.75 μmol TE/g, 3343.76 to 4131.49 μmol TE/g, and 436.20 to 481.62 μmol TE/g, as determined by DPPH, FRAP, ORAC, and PCL, respectively [144]. The aqueous extract from MO acts as a potent free radical scavenger [148], while the ethanolic stem extract protects against epidermal oxidative stress injury induced by H_2_O_2_ in keratinocytes [149]. Similarly, methanol extracts from MO leaves were found to protect against H_2_O_2_-induced oxidative damage by improving cell viability, reducing free radicals, and enhancing antioxidant defenses, including increased glutathione (GSH) levels and antioxidant enzyme activity. The extracts also prevented mitochondrial dysfunction by regulating calcium levels and improving mitochondrial membrane potential [150].

Alcoholic extracts also mitigated glucose-induced cataractogenesis in goat eye lenses by regulating GSH levels [151]. Isoquercetin, astragalin, and crypto-chlorogenic acid extracted from MO leaves reduced reactive oxygen species (ROS) in HEK-293 cells and exhibited liver-protective effects against diclofenac sodium-induced toxicity [152]. Se-enriched Moringa oleifera seed protein hydrolysate demonstrated strong cellular protection, increased cell viability, reduced ROS accumulation, and enhanced the activity of antioxidant enzymes like superoxide dismutase (SOD) and catalase (CAT) in HepG2 cells [153].

Inflammation is a protective response, but it can also contribute to chronic diseases such as colitis, diabetes, and arthritis. While long-term use of anti-inflammatory drugs may harm organ function, non-pharmacological treatments like herbal therapies are gaining attention. MO has demonstrated anti-inflammatory potential due to its rich composition of tannins, triterpenoids, saponins, flavonoids, alkaloids, and phenols. Furthermore, its high levels of vitamins A, C, and E; carotenoids; and polyphenols provide antioxidant, anti-inflammatory, and antibacterial benefits. The flavonoid content in MO makes it effective for treating and preventing conditions such as gingivitis and periodontal diseases [154].

Aurnatiamide acetate and 1,3-dibenzyl urea from MO roots inhibit TNF-α (tumor necrosis factor-α) production. At the same time, its fruit extract prevents NF-κB (nuclear factor kappa B) translocation, though the chloroform extract is cytotoxic at high concentrations [155]. MO’s anti-inflammatory effects are linked to reduced levels of IL-1, IL-6, IL-10, TNF-α, prostaglandin E2, and COX-2 (cyclooxygenase-2) [156].

MO seed extract shows therapeutic potential for inflammatory bowel diseases (IBD) by reducing ulcerative colitis severity, improving disease activity scores, and decreasing pro-inflammatory cytokines. Compounds like kaempferol, astragalin, and KETTTIVR contribute to its antioxidant and anti-inflammatory properties, helping restore gut microbiota balance and enhance intestinal barrier function [157].

Nizazirin, found in MO seeds, plays a key role in its anti-inflammatory effects. MO seed extracts prevent protein denaturation and contain flavonoids, phenolic acids, and glucosinolates. MIC-1, a glucosinolate, exhibits strong anti-inflammatory properties and high bioavailability. MO seed oil also reduces inflammatory markers in colitis, lung damage, and arthritis, supporting its potential for managing inflammatory conditions [118].

MO benefits CDs like diabetes, cardiovascular diseases, arthritis, and cancer by improving insulin sensitivity, reducing blood glucose levels, and protecting against hyperglycemia-induced organ damage [158,159]. Its antioxidant properties enhance intracellular defenses, including CAT and SOD, mitigating oxidative stress [158,159]. MO extracts also regulate inflammatory cytokines, boost antioxidant enzyme activity, and inhibit NF-κB, which are crucial in combating inflammation and degenerative diseases [120,160]. Additionally, it improves lipid metabolism, stimulates insulin release, and enhances glucose uptake, aiding in diabetes and metabolic syndrome management [119,161].

MO compounds such as quercetin and kaempferol inhibit inflammatory enzymes like cyclooxygenase and lipoxygenase [157]. Its anti-inflammatory effects are enhanced when combined with GABA, which further suppresses inflammatory pathways [162]. Isothiocyanates in MO regulate signaling pathways, reducing TNF-α and IL-1β levels [157,162]. MO may also support conditions like IBD and non-alcoholic fatty liver disease [157] and demonstrates anti-arthritic and skin anti-inflammatory effects [122,163]. It significantly reduces inflammatory markers, including IL-1, IL-6, TNF-α, and COX-2, which are linked to CDs [156,164], and suppresses macrophage-mediated inflammation, highlighting its role in reducing chronic inflammation [165].

### 4.3. Moringa Phytochemicals Enhance Metabolic Functions for Managing CDs and Related Conditions

Obesity induces oxidative stress, disrupting the balance between pro-oxidants and antioxidants, leading to increased fat accumulation, insulin resistance, and inflammation, particularly in visceral fat, contributing to dyslipidemia and obesity-related complications [118]. MO seed oil extract (800 mg/kg b.w.) and lycopene (LYC) significantly improved high-fat diet (HFD)-induced metabolic disturbances in Sprague–Dawley rats by enhancing antioxidant enzymes, reducing lipid peroxidation and inflammatory cytokines, and decreasing iNOS (inducible nitric oxide synthase) protein expression, demonstrating anti-obesity potential [166]. Similarly, a 70% ethanol extract of MO leaves (MO 400) reduced body weight, adiposity, glucose, insulin, and HOMA-IR (homeostatic model assessment for insulin resistance) while increasing insulin sensitivity. MO 400 also suppressed FAS (fatty acid synthase) and HMG-CoA reductase (3-hydroxy-3-methylglutaryl-CoA reductase) while enhancing MC4R (3-hydroxy-3-methylglutaryl-CoA reductase) and PPAR-α (peroxisome proliferator-activated receptor alpha) expression. A clinical study showed that MO 400 reduced BMI (body mass index), total cholesterol, and LDL (low-density lipoprotein) in obese patients, suggesting its effectiveness in obesity and metabolic disorder management [167].

*Moringa peregrina* (MP) leaf extracts also mitigated high-fat diet (HFD)-induced obesity in rats, reducing food intake, weight gain, fat deposition, plasma glucose, insulin, and leptin levels while improving glucose tolerance. MP extracts lowered serum cholesterol, triglycerides, and LDL while increasing HDL (high-density lipoprotein) and fecal lipid excretion. Additionally, they improved liver function, reduced hepatic fat accumulation, and increased antioxidant enzyme activity, particularly at a 600 mg/kg dose [168].

At a molecular level, MO leaf extract regulates lipid metabolism via yes-associated protein (YAP) and the transcriptional coactivator with PDZ-binding motif (TAZ) (YAP/TAZ) signaling pathway [169]. MO powder (MOP) supplementation protected against HFD-induced obesity in mice by preventing weight gain, improving lipid profiles, and modulating gut microbiota, increasing beneficial bacteria (*Bacteroides*, *Norank_f_Ruminococcaceae*, and *Oscillibacter*) while reducing obesity-associated bacteria (*Blautia*, *Alistipes*, and *Tyzzerella*), suggesting prebiotic potential [170].

MO seeds contain bioactive compounds such as flavonoids, phenolic acids, and alkaloids that help reduce weight and obesity-related complications. Methanolic MO seed extracts (MOSE) significantly decreased BMI, body fat, and LDL while increasing HDL in obese rats, showing lipid-lowering effects. MO seed oil also reduced non-esterified fatty acids, dyslipidemia, and hyperglycemia in HFD-fed rats, attributing its anti-obesity effects to phytochemicals like phenolic acids, flavonoids, and saponins [118].

MO’s role in improving metabolic function and mitigating metabolic syndrome factors, such as obesity and hyperlipidemia, supports its potential as a natural therapeutic agent [161,171]. MO bioactive compounds, especially isothiocyanates, regulate lipid metabolism, reduce body weight, and enhance insulin sensitivity, crucial for managing obesity-related conditions like diabetes and cardiovascular diseases [168,171,172,173]. MO has been shown to improve metabolic syndrome indices, including hyperglycemia, hypertension, and dyslipidemia, reducing the risk of diabetes and cardiovascular diseases [161]. Additionally, MO influences genes related to adipogenesis, glucose uptake, and insulin resistance [172]; lowers oxidative stress and inflammation linked to obesity [166,168,174]; and improves glucose tolerance and insulin signaling, thereby reducing type 2 diabetes risk [174]. MO polysaccharides also promote beneficial gut microbiota, further supporting weight management and metabolic health [170].

### 4.4. Anti-Diabetic Properties of Moringa and Its Potential to Alleviate Neuropathic Pain in Diabetic Neuropathy

Diabetes mellitus (DM) is a metabolic disorder characterized by hyperglycemia, leading to complications such as retinopathy, neuropathy, and nephropathy. MO has demonstrated potential in managing diabetes, particularly in alleviating diabetic neuropathic pain. Studies on diabetic rats with chronic constriction-induced neuropathy showed that MO leaf extract significantly reduced pain symptoms. MO’s high phenolic acid content contributes to its protective effects against oxidative stress by scavenging free radicals and lowering inflammatory cytokine production [175].

Human studies suggest that MO may reduce glucose levels by enhancing insulin secretion and sensitivity while inhibiting amylase and glucosidase activities. Animal studies propose additional mechanisms, including increased glucose uptake in the muscles and liver, inhibition of intestinal glucose absorption, and reduced hepatic gluconeogenesis [176]. While human trials have shown promising but inconsistent results, some studies reported reduced postprandial glucose and HbA1C (hemoglobin A1C) levels following MO supplementation. For instance, an 8 g daily intake of MO powder led to a 26% decrease in postprandial glucose, while another study observed a 29% reduction over three months. However, methodological limitations, including small sample sizes and baseline discrepancies, affect the reliability of these findings. Some studies also noted improvements in cholesterol levels, antioxidant vitamins, and oxidative stress markers. In a placebo-controlled study, no significant changes in glucose or HbA1C were observed, but insulin secretion increased with MO use. Further large-scale clinical trials are needed to confirm its efficacy as an adjunct therapy for hyperglycemia [177].

In streptozotocin-induced diabetic rats, MO leaf treatment (150 mg/kg and 300 mg/kg) led to reduced liver glutathione peroxidase (GSH-Px) levels but increased superoxide dismutase (SOD) levels, with notable improvements in liver catalase (CAT) at the higher dose. These findings suggest that MO helps mitigate oxidative stress and kidney and liver damage in diabetes [178]. Additionally, oral administration of MO methanolic extract from flowering buds (500 mg/kg) significantly lowered blood glucose levels in diabetic mice while improving body weight, showing effects comparable to Glibenclamide (10 mg/kg) [179]. MO leaf extract-mediated gold nanoparticles (MO-AuNPs) exhibited potent antioxidant and antidiabetic effects in a concentration-dependent manner [180].

MO has also shown anti-diabetic nephropathy effects in streptozotocin-induced diabetic rats. The extract lowered blood glucose, improved kidney function, and reduced oxidative stress. Histological analysis indicated reduced glomerular basement membrane thickening and alleviation of pathological changes. These effects were linked to downregulation of TGF-β1 (transforming growth factor beta 1) and type IV collagen genes. A methanolic extract combining MO leaves and seeds significantly improved fasting blood glucose, cholesterol, triglycerides, creatinine, liver enzymes, and oxidative stress markers in alloxan-induced diabetic mice [181,182].

MO extracts, particularly from seeds, have been effective in treating insulin-resistant Type 2 diabetes and streptozotocin-induced Type 1 diabetes in animal models. MO seed powder reduced fasting blood glucose while increasing antioxidant enzyme activity, potentially lowering ROS production in beta cells. Antioxidants such as quercetin in MO help scavenge ROS, protecting beta cells and improving hyperglycemia. Further studies showed that MO seed flour significantly reduced lipid peroxidation and enhanced antioxidant enzyme levels in blood and kidney tissues. MO also improved immune function, lowered inflammatory markers, and improved fasting blood sugar and glycosylated hemoglobin levels. MO supplementation enhanced kidney function and electrolyte balance in diabetic rats. Bioactive compounds such as flavonoids may contribute to their anti-diabetic properties, with computational studies suggesting potential interactions with mutant insulin receptors, indicating a possible mechanism for diabetes treatment. Additionally, MO seed oil aids in wound healing, particularly in diabetic conditions. While MO shows significant promise in glycemic control and wound healing, further research is needed to elucidate its bioactive components and therapeutic applications [118].

### 4.5. Wound-Healing Properties for Alleviating the Consequences of CDs

Topical administration of MO extract on excision wounds significantly accelerated wound healing compared to untreated and MEBO^®^-treated groups. MO increased the expression of TGF-β1, VEGF (vascular endothelial growth factor), and Type I collagen while reducing inflammatory markers like IL-1β and TNF-α. In vitro, antioxidant assays revealed vigorous free radical scavenging activity. In silico studies showed that MO seed metabolites could bind to the active sites of proteins related to wound healing, such as TNF-α, TGFβR1 (transforming growth factor beta receptor 1), and IL-1β. Quercetin, caffeic acid, and kaempferol exhibited the highest binding with these proteins. In vitro tests confirmed the effectiveness of quercetin, kaempferol, and caffeic acid, with kaempferol showing the highest activity in inhibiting IL-6 and matrix metallopeptidases (MMPs). Thus, MO seeds are a promising source of bioactive compounds for antioxidant and wound-healing purposes [183]. MO exhibits significant wound-healing properties, making it beneficial for managing chronic conditions like diabetic ulcers. MO is rich in bioactive compounds, including flavonoids, phenolic acids, and vitamins, which contribute to its antioxidant, anti-inflammatory, and antimicrobial activities [5,184]. MO extracts demonstrate antibacterial efficacy against pathogens such as *Staphylococcus aureus* and *Pseudomonas aeruginosa*. At the same time, their potent antioxidant properties help mitigate oxidative stress, a major factor in delayed wound healing in diabetic patients [185,186]. Additionally, MO promotes collagen synthesis and accelerates tissue regeneration, reducing healing time in chronic wounds [183,184,187]. MO extracts also enhance cell migration and proliferation, essential processes for wound closure and healing, particularly in diabetic conditions [185,186].

MO seed oil (MOSO) and oleic acid (OA) were found to accelerate wound healing in healthy mice. In contrast, in immunosuppressed and diabetic mice, healing was delayed but improved with MOSO and OA treatments. These treatments enhanced collagen content and restored myofibroblast levels, which are crucial for wound repair. OA specifically increased collagen in healthy wounds, suggesting MOSO’s potential to accelerate acute and chronic wound healing by boosting collagen synthesis and myofibroblast activity. Further research is needed to elucidate the underlying mechanisms [187].

The hydroalcoholic extract of MO seeds demonstrated strong antioxidant and antimicrobial properties, confirmed via agar well diffusion assays. Hydrogels formulated with MO seed extract exhibited superior wound healing in excision wound models, outperforming control and standard treatments up to the 13th day. The hydrogel significantly enhanced wound-breaking strength in incision models, with histopathological analysis further validating its wound-healing efficacy [188].

Most studies on MO’s wound-healing effects focus on topical applications, including gels, pastes, patches, film dressings, and ointments. Limited research has explored oral administration of aqueous extracts, with only a few studies combining both methods. However, only 28% of the studies verified the plant through voucher specimen deposition, and none conducted qualitative or quantitative phytochemical analysis. Despite these standardization challenges, MO’s wound-healing and antidiabetic properties suggest it may be an effective treatment for diabetic wounds. MO has been shown to enhance wound healing and improve hyperglycemia in diabetic animal models, likely due to bioactive compounds such as gallic acid, rutin, vicenin-2, flavonoids, and phenolic metabolites. Given that diabetic patients have an 11% higher risk of wound infections, often caused by *Escherichia coli*, *P. aeruginosa*, and *S. aureus*, MO’s antimicrobial and wound-healing properties highlight its therapeutic potential [189].

Topical administration of MO extract significantly accelerated wound healing in excision wounds compared to untreated and MEBO^®^-treated groups. MO treatment increased the expression of TGF-β1, VEGF (vascular endothelial growth factor), and Type I collagen while reducing inflammatory markers like IL-1β and TNF-α. Antioxidant assays revealed strong free radical scavenging activity. In silico studies indicated that MO seed metabolites could bind to active sites of key wound-healing proteins, including TNF-α, TGFβR1, and IL-1β. Quercetin, caffeic acid, and kaempferol showed the highest binding affinity to these proteins, with in vitro studies confirming their effectiveness in inhibiting IL-6 and matrix metallopeptidases (MMPs). Kaempferol exhibited the strongest inhibitory activity, highlighting MO seeds as a promising source of bioactive compounds for antioxidant and wound-healing applications [183].

### 4.6. Cardioprotective Properties for Managing Hypertension and Related Conditions

MO seeds and leaves exhibit significant cardioprotective properties, primarily due to their high antioxidant content, which helps reduce oxidative stress—a major factor in hypertension and heart failure. Oleic acid in MO seeds further contributes to lowering blood pressure and reducing cardiovascular risk [118].

Quercetin, a bioactive compound found in MO leaf extract, has demonstrated protective effects against dyslipidemia and atherosclerosis in a rat model. In rats fed a high-fat diet for 10 weeks, quercetin (25 mg/kg body weight) significantly reduced malondialdehyde (MDA) levels, indicating lower oxidative stress. Additionally, it decreased intercellular adhesion molecule (ICAM) and C-reactive protein (CRP) levels, suggesting reduced inflammation. Although LDL levels increased with quercetin administration, histological analysis revealed significant improvements in coronary and carotid artery structure, indicating protection against atherosclerosis. These findings suggest quercetin from MO leaves may serve as a potential therapeutic agent for cardiovascular diseases [190].

The cardioprotective effects of MO leaf extract were also evaluated against cisplatin-induced cardiotoxicity in rats. Rats receiving MO extract (250 mg/kg and 500 mg/kg) alongside cisplatin (10 mg/kg) showed improved cardiac structure, reduced injury markers, and enhanced antioxidant defenses in a dose-dependent manner. The extract significantly mitigated oxidative stress and lipid profile disruptions, suggesting its potential for reducing cisplatin-induced heart damage [191].

Similarly, ethanolic MO leaf extract (MOE) formulated into niosomes (MOE-NIO) was tested against doxorubicin (DOX)-induced cardiotoxicity in rats. Pre- and co-treatment with MOE-NIO significantly reduced serum levels of cardiac injury markers, including AST, CK-MB (creatine kinase-myocardial band), LDH (lactate dehydrogenase), and cTnI (cardiac troponin I). It also lowered inflammatory markers like myeloperoxidase (MPO) and TNF-α while inhibiting NF-κB and p38 MAPK (p38 mitogen-activated protein) activation, which are associated with inflammation and apoptosis. Additionally, MOE-NIO reduced lipid peroxidation, enhanced antioxidant enzyme activity (SOD and glutathione), and restored Nrf2 expression, a key regulator of the antioxidant response. These findings suggest MOE-NIO could serve as a cardioprotective agent for patients undergoing DOX-based cancer treatment [113].

MO’s cardioprotective effects are attributed to its bioactive compounds, including flavonoids, isothiocyanates, and vitamins, which reduce inflammation and oxidative stress and improve lipid and glucose metabolism—key factors in managing cardiovascular diseases (CVDs) [5,192,193]. MO modulates these effects by suppressing NF-κB and enhancing antioxidant pathways like Nrf2/Keap1 [113,192,194]. Additionally, it reduces drug-induced cardiotoxicity, as seen in DOX treatments, by lowering oxidative stress, inflammation, and apoptosis while enhancing antioxidant defenses [113,195]. MO, when combined with treatments like acarbose, has shown synergistic effects in managing diabetic cardiomyopathy by improving biochemical pathways and antioxidant status [196].

MO also contains bioactive compounds such as niazicin-A, niazimin-A, and niaziminin-B, which inhibit angiotensin-converting enzyme (ACE), a key regulator of blood pressure. These compounds exhibit strong ACE-binding affinity, potentially surpassing standard drugs like Captopril and Enalapril, suggesting their potential as natural ACE inhibitors [197,198]. Peptides derived from MO, such as Leu-Gly-Phe-Phe (LGF) and Gly-Leu-Phe-Phe (GLFF), have demonstrated dual inhibitory effects on ACE and renin, significantly reducing blood pressure in hypertensive rat models [199]. Additionally, MO extracts, both ethanolic and aqueous, have been shown to lower systolic and diastolic blood pressure in spontaneously hypertensive rats, likely due to their diuretic and ACE-inhibitory activities [200,201].

### 4.7. Immunomodulatory Properties of Moringa for Alleviating CDs

MO exhibits immunosuppressive and immunomodulatory properties that can aid in regulating immune responses in various conditions, including autoimmune diseases, asthma, and ulcerative colitis [202,203]. Its methanolic extract contains bioactive compounds such as isothiocyanates and glycoside cyanide, which have immunostimulatory effects and enhance immune function. These compounds have been used to manage immune-related disorders, including cancer, hypertension, and diabetes, thereby strengthening host immunity [204,205]

In a study involving autoimmune patients who consumed 2 g of MO leaf extract daily for four weeks, significant reductions in mean platelet volume (MPV) and neutrophil-to-lymphocyte ratio (NLR) were observed, indicating a decrease in inflammation [206]. Additionally, HIV-positive individuals supplemented with MO while on antiretroviral therapy experienced increased CD4-cell levels and decreased TNF-α levels. MO supplementation also improved hematological abnormalities such as anemia, thrombocytopenia, leukopenia, lymphopenia, and neutropenia [207].

MO has also shown potential in managing atopic dermatitis (AD). In a mouse model with 2,4-dinitrochlorobenzene (DNCB)-induced AD-like symptoms, treatment with *Moringa concanensis* inhibited inflammatory mediators and pro-inflammatory cytokines like IL-1β. In human epidermal keratinocyte cells (HaCaT), *M. concanensis* downregulated NLRP3 (NLR family pyrin domain containing 3) inflammasome activation and inhibited the activation of JNK (c-Jun N-terminal kinase), AP-1 (activator protein 1), and p65, which are associated with inflammation. Mice treated with *M. concanensis* showed improved clinical symptoms, including reduced skin inflammation, dermatitis scores, and trans-epidermal water loss (TEWL), suggesting its effectiveness in alleviating AD through NLRP3 inflammasome inhibition [208].

MO has also demonstrated benefits for thyroid health. Patients who consumed 5 g of fresh MO leaves twice daily for 45 days showed significant reductions in serum thyroid-stimulating hormone (TSH) levels and increased levels of triiodothyronine (T3) and thyroxine (T4), indicating its potential for managing primary hypothyroidism [209].

In an albino rat model, administration of MO leaf aqueous extract (800 mg/kg or 1600 mg/kg) significantly increased white blood cell counts after 14 days, suggesting that MO may help prevent immune deficiency [210]. Additionally, rats treated with MO extract (300 mg/kg) after pilocarpine administration exhibited improved hippocampal structure, indicating its potential to reverse hippocampal sclerosis in temporal lobe epilepsy [211].

#### Anti-Arthritis Properties

MO has shown promising anti-arthritis effects. In patients with rheumatoid arthritis (RA) treated with 40.50 mg/kg BW/day of MO extract for one month, IL-6 levels and Simplified Disease Activity Index (SDAI) scores significantly decreased, demonstrating MO’s effectiveness in reducing inflammation and disease activity in RA [212].

Molecular docking studies further support MO’s anti-arthritic potential. Quercetin and niazirinin, key compounds in MO, were tested against the GLS-1 (glutaminase) protein, showing strong binding affinities of −4.8 and −5.94 Kcal/mol, respectively, indicating their potential role as anti-arthritic agents [213]. Similarly, molecular modeling assessed quercetin and niazirinin against the HK-2 (hexokinase 2) protein, with binding energies of −6.66 and −5.58 Kcal/mol, respectively, suggesting their potential in arthritis treatment [214].

### 4.8. Anti-Ulcer and Gastroprotective Properties to Manage Chronic Gastrointestinal Diseases

MO demonstrates significant anti-ulcer and gastroprotective properties, making it a promising natural remedy for chronic gastrointestinal diseases. Studies on aspirin-induced gastric ulcers in rats have shown that MO leaves and extracts effectively reduce ulcer index, gastric volume, and total acidity. Additionally, they enhance gastric mucosal mucin content and help normalize plasma nitric oxide levels, which play a crucial role in gastric protection [215,216].

A separate study examined the combined effects of aqueous extracts from *Vernonia amygdalina* (bitter leaf) and *M. oleifera* on gastric ulcer healing in rats. The rats were divided into seven groups, with one serving as a control and the others receiving indomethacin to induce gastric ulcers. Various treatments, including omeprazole and herbal extracts, were administered for 21 days, after which gastric tissues were analyzed. The combination of *V. amygdalina* and MO significantly improved gastric mucosal healing. Histological analysis revealed enhanced tissue integrity and reduced ulcer indices, suggesting a synergistic effect between the two herbs. These findings indicate that MO, especially in combination with *V. amygdalina*, could be an effective dietary therapy for gastric ulcers, offering a natural alternative to conventional treatments for gastrointestinal disorders [217].

### 4.9. Antipyretic and Antinociceptive Properties for Alleviating Pain in CDs

MO possesses notable antipyretic and antinociceptive properties, making it a promising natural remedy for pain management in CDs. Studies have demonstrated its analgesic effects in various animal models, including formalin and hot-plate tests. In a study using *Mus musculus*, MO leaf extract significantly reduced pain-induced writhing responses, comparable to aspirin, confirming its pain-relieving potential [115]. Another study on Swiss Webster rats found that MO leaf extract, when combined with *Phyllanthus niruri* (meniran) extract, exhibited analgesic effects similar to diclofenac sodium, highlighting its efficacy as a natural analgesic [218].

Additionally, in male rats, MO extract at doses of 200 and 300 mg/kg significantly reduced pain responses in multiple tests, with the highest dose showing the most potent effects. The extract demonstrated both central and peripheral analgesic activity, reinforcing its potential as a therapeutic agent for pain relief [219].

### 4.10. Antidepressant Properties for Alleviating Symptoms of CDs

MO demonstrates potential antidepressant properties, likely mediated through noradrenergic–serotonergic neurotransmission, similar to selective serotonin reuptake inhibitors (SSRIs) [94]. MO may also exert anxiolytic-like effects via GABA mimetic action [220]. In patients with rheumatoid arthritis, MO leaf extract significantly reduced depression scores and serum cortisol levels, suggesting its role as a complementary therapy for depression in chronic diseases [221].

Ethanolic MO leaf extract exhibited antidepressant effects in mice, as shown by the forced swim test (FST), tail suspension test (TST), and locomotor activity test (LAT). When combined with fluoxetine (10 mg/kg), MO (200 mg/kg) enhanced antidepressant effects, possibly due to modulation of the noradrenergic–serotonergic pathway and anti-inflammatory properties [94].

Additionally, MO seed lectin (WSMoL) demonstrated antidepressant-like effects through monoaminergic signaling. In TST, WSMoL combined with fluoxetine (5 mg/kg) significantly reduced immobility time, with its effects blocked by monoaminergic pathway inhibitors, confirming its role in neurotransmitter modulation [222].

In pregnant women with hypertension, nano acupressure particles of MO extract (500 mg/day) reduced anxiety and increased serotonin levels, potentially influencing blood pressure regulation through improved mood [223].

Further, WSMoL was tested in mice subjected to unpredictable chronic mild stress (UCMS). Treatment with WSMoL (2 or 4 mg/kg) for 21 days significantly reduced anxiety and depression-like behaviors, decreased pro-inflammatory cytokines (IL-2, IL-6, and TNF-α), and increased brain levels of dopamine, serotonin, and noradrenaline, suggesting MO alleviates stress-induced mood disorders by reducing neuroinflammation and modulating brain monoamines [224].

### 4.11. Neuroprotective Properties and Neuropharmacological Activity for Managing CDs

MO contains phenolic compounds, including flavonoids and isothiocyanates, that provide neuroprotective effects against oxidative stress, neurodegenerative disorders, and liver disease [119,120]. These bioactive compounds also help reduce DNA damage and inhibit inflammatory pathways, contributing to the prevention of CDs [119,160]. MO’s phytochemical composition, rich in flavonoids, phenolic acids, and thiocyanates, supports its potential in managing neurodegenerative conditions like Alzheimer’s, Parkinson’s, and Huntington’s diseases by modulating neurotransmitter levels and slowing disease progression [117,225]. Its neuroprotective mechanisms involve key molecular pathways, such as the NF-kB/Nrf2/HO-1 signaling pathway, which maintains mitochondrial function and promotes neurogenesis [226]. Phytochemicals like moringin, astragalin, and isoquercitrin further enhance its neuroprotective properties by reducing neuroinflammation and oxidative stress [227,228]. Niazimicin, a thiocarbamate glycoside in MO seeds, has demonstrated neuroprotective effects by lowering oxidative stress markers and improving cognitive function in experimental models [229]. Additionally, MO root extract exhibited sedative effects, increasing sleep duration in mice treated with pentobarbital sodium and diazepam [230].

MO supplementation (1%, 5%, and 10% of the diet) improved cognitive function in a study where it increased acetylcholine levels in the hippocampus by inhibiting acetylcholinesterase activity, thereby reversing scopolamine-induced memory deficits. Additionally, aqueous MO extracts (300 and 500 mg/kg) significantly elevated dopamine and serotonin levels in rats, with stronger effects at higher doses, suggesting its role in neurotransmitter regulation and neuroprotection [231].

A comprehensive review of 120 studies highlighted MO’s neuroprotective and antioxidant properties, linking its phytochemicals—alkaloids, flavonoids, phenolics, terpenoids, steroids, glycosides, saponins, and coumarins—to potential applications in treating neurodegenerative diseases and cognitive impairments. Research models using rodents (Wistar and Swiss Albino rats) and human cell lines (SH-SY5Y and PDLSCs) have been developed to investigate MO’s neuroprotective effects. Studies often induce cognitive deficits using scopolamine, aluminum chloride, or insecticides, which disrupt cholinergic signaling and reduce key proteins like CREB (cAMP-response element-binding protein) and BDNF (brain-derived neurotrophic factor (BDNF). The SH-SY5Y neuroblastoma cell line, widely used for neuroprotection studies, mimics human neuron behavior, while mesenchymal stem cells (e.g., PDLSCs) show promise for neurodegeneration therapy [232].

MO seed extracts also demonstrated protective effects in a Parkinson’s disease (PD) mouse model induced by rotenone. Mice treated with aqueous and ethanolic MO seed extracts showed improved motor function, increased antioxidant activity, and reduced neurodegeneration. These findings suggest that MO seed extracts may offer therapeutic potential for PD and other neurodegenerative disorders [233].

In oxidative stress-induced SH-SY5Y neuroblastoma cells, MO leaf extract reduced ROS levels while enhancing antioxidant enzyme activities (GSH-Px, CAT, and SOD). MO treatment downregulated apoptotic genes (Bax and Caspase-3) and upregulated neuroplasticity markers (BDNF), as well as proteins involved in neuronal signaling (p-Akt and p-CREB), reinforcing its neuroprotective role through antioxidant and antiapoptotic mechanisms [234]. Furthermore, MO leaf extract showed neuroprotective effects in a rat model of monosodium glutamate (MSG)-induced cerebellar ataxia via the Nrf2-Keap1 pathway. In this study, MO improved motor function, preserved Purkinje cells, increased GSH levels, and elevated Nrf2 expression. Prevention therapy with MO demonstrated stronger benefits than treatment therapy, highlighting its potential to mitigate MSG-induced neurotoxicity [235].

### 4.12. Hepatoprotective Properties for Alleviating CDs

MO exhibits hepatoprotective properties, making it a potential candidate for managing chronic liver diseases. Its beneficial effects are attributed to its antioxidant and anti-inflammatory activities, which mitigate oxidative stress and inflammation—common pathways in liver damage [168,193]. MO extracts have been shown to significantly lower liver enzyme levels and improve liver histology in animal models of toxin-induced liver injury, such as those caused by carbon tetrachloride and lead acetate [168,236,237]. Additionally, MO extracts have reduced liver damage markers in models of drug-induced hepatotoxicity [168,238].

In a study on female albino mice with polycystic ovary syndrome (PCOS), MO leaf powder (MOLP) and ethanol leaf extract (MOLE) significantly reduced liver and kidney dysfunction markers, including ALT (alanine aminotransferase), AST (aspartate aminotransferase), ALP (alkaline phosphatase) total bilirubin, urea, and creatinine while increasing total protein, albumin, globulin, and the albumin/globulin (A/G) ratio. MO also reduced oxidative stress, as measured by MDA levels, indicating an overall improvement in liver and kidney function in PCOS-induced mice [239].

MO was also studied in male Wistar rats for its potential hepatoprotective and hypolipidemic properties when combined with *Linum usitatissimum* seed extracts. In a diabetes model induced by streptozotocin, the extract mixture significantly reduced lipid levels, urea, creatinine, and liver function markers, suggesting its effectiveness in preventing long-term complications of diabetes [240].

Green-synthesized ZnO nanoparticles (ZnO-NPs) derived from MO leaves exhibited significant hepatoprotective effects by restoring liver and kidney function, enhancing antioxidant enzyme activity, and improving hematological parameters. These effects were more pronounced than those of the aqueous MO leaf extract alone, indicating the superior efficacy of ZnO-NPs in drug delivery mechanisms [241].

Due to the poor water solubility of MO’s bioactive compounds, a nanosuspension formulation was developed to improve its bioavailability. The nanosuspension demonstrated faster dissolution, a 1.19-fold increase in bioavailability, and superior antioxidant and hepatoprotective potential compared to the crude extract, suggesting an optimized formulation for enhanced therapeutic efficacy [242].

Additionally, aqueous *M. oleifera* Lam. leaf extract (AMOLE) was evaluated for its protective effects against cadmium (Cd) toxicity in human HepG2 hepatocytes. Cd, a toxic pollutant that accumulates in the liver, induces oxidative stress and hepatotoxicity. Pretreatment with AMOLE significantly reduced Cd-induced oxidative stress. Metabolomic analysis identified phenolic and carboxylic acids, including quercetin, as major antioxidants responsible for its protective effects. AMOLE’s hepatoprotective mechanisms involve free radical elimination, upregulation of antioxidant defenses via the Nrf2 pathway, increasing GPx1 (glutathione peroxidase 1) and HO-1 (Heme oxygenase 1) expression and suppressing Cd uptake, indicating its potential for clinical applications in Cd toxicity prevention [243].

### 4.13. Properties Against Non-Alcoholic Fatty Liver Disease (NAFLD)

Non-alcoholic fatty liver disease (NAFLD) includes non-alcoholic fatty liver (NAFL) and non-alcoholic steatohepatitis (NASH), with the latter increasing the risk of cirrhosis and liver failure. NAFLD is linked to metabolic disorders like obesity, dyslipidemia, and type 2 diabetes, with genetic factors also contributing. MO has shown potential in mitigating NAFLD by reducing liver fat accumulation, lowering cholesterol and triglycerides, and decreasing inflammation. Its bioactive compounds, including quercetin, chlorogenic acid (CGA), and niazirin, regulate lipid metabolism and inflammation. CGA inhibits fatty acid synthase and enhances β-oxidation via PPARα activation. MO also reduces pro-inflammatory cytokines and NF-κB signaling while improving antioxidant status. Fermented MO extract further supports liver health by enhancing AMPK phosphorylation, promoting lipolysis, and reducing lipogenesis, slowing NAFLD progression [157].

1-phenyl-2-pentanol (1-PHE), a compound derived from MO leaves, has demonstrated anti-fibrotic effects in human hepatic stellate cells (LX-2) activated by TGF-β1. Treatment with 1-PHE downregulated fibrosis markers such as COL1A1 (collagen type I alpha one chain), COL4A1 (collagen type IV alpha one chain), SMAD2/3 (mothers against decapentaplegic homologs 2 and 3 proteins), and MMP2 (matrix metalloproteinase-2) while reducing MMP-9 secretion. Proteomic analysis suggests that 1-PHE inhibits the TGF-β1 and Wnt/β-catenin pathways, reducing hepatic stellate cell activation and indicating potential therapeutic applications for liver fibrosis [244].

MO has also been studied for its hepatoprotective effects against paracetamol-induced liver toxicity, a major cause of acute liver failure. It has shown antioxidant, tissue-protective, analgesic, antihypertensive, and immunomodulatory properties, mitigating paracetamol toxicity in male rats [245].

### 4.14. Nephroprotective Properties for Alleviating CDs

MO exhibits nephroprotective potential in chronic kidney diseases due to its phenolic compounds that provide antioxidant, anti-inflammatory, and anti-apoptotic benefits. It enhances antioxidant enzyme activity (SOD, CAT, and GSH) to reduce oxidative stress in kidney tissues and downregulates inflammatory cytokines such as TNF-α, IL-1β, and IL-6, thereby mitigating kidney inflammation and damage [159,246,247,248]. MO also regulates apoptotic proteins to prevent cell death in kidney tissues, proving beneficial in lead-induced nephrotoxicity [247,248].

MO has demonstrated efficacy in protecting against nephrotoxicity caused by environmental toxins (e.g., lead) and drugs (e.g., gentamicin) by restoring redox balance and reducing inflammation and apoptosis [247,248,249]. Selenium nanoparticles (SeNPs) synthesized using ethanolic MO leaf extract (MOLE) were tested for nephroprotective effects against melamine-induced nephrotoxicity in male Sprague–Dawley rats. Both MOLE and MOLE-SeNPs mitigated oxidative stress, improved histopathological alterations, and modulated apoptosis-related genes. The ethanolic MOLE extract exhibited stronger protective effects than the MOLE-SeNP conjugate, highlighting MO’s role in counteracting melamine-induced renal damage [250].

MIC-1, another MO-derived compound, has shown potential in improving renal function and reducing kidney tissue injury in db/db mice. MIC-1 reduced oxidative stress markers (ROS and MDA) while enhancing antioxidants (GSH, SOD, and CAT). It activated the ERK/Nrf2/HO-1 signaling pathway, increasing levels of p-ERK, Nrf2, SOD-1, and HO-1, while suppressing NF-κB signaling and reducing p-IKBα (nuclear factor of kappa light polypeptide gene enhancer in B-cells inhibitor alpha) and p-NF-κB (P65/P65) levels. Additionally, MIC-1 improved podocyte marker expression (podocalyxin and synaptopodin), suggesting its role in preserving kidney tissue integrity in diabetic nephropathy [251].

MO was also found effective in mitigating renal ischemia/reperfusion (I/R) injury, which causes significant kidney damage, oxidative stress, inflammation, and apoptosis. A MO-based feed supplement significantly reduced kidney dysfunction markers (serum creatinine and BUN), oxidative stress (MDA and hydrogen peroxide), inflammation markers (TNF-α and IL-6), and pro-apoptotic proteins (Bax and Caspase-3), indicating its protective role in renal I/R injury through oxidative stress and apoptosis regulation [252].

### 4.15. Anti-Tumoral Properties

Developing anticancer drugs from natural sources like MO offers a cost-effective treatment approach. Rutin, identified via computational modeling, exhibits a strong binding affinity to BRCA-1 (breast cancer gene-1) [68], while MO-AuNPs demonstrate potent anticancer activity with an IC_50_ value of 67.92 μg/mL [180]. MITC-12 (4-[(α-L-rhamnose oxy) benzyl] isothiocyanate), a derivative of MITC, inhibits various cancer cell lines, including U251 (human glioblastoma astrocytoma), A375 (human malignant melanoma), A431 (human squamous carcinoma), HCT-116 (human colon cancer), HeLa (human cervix epithelioid carcinoma), and MDA-MB-231 (human Caucasian breast adenocarcinoma). MITC-12 was shown to reduce U251 cell proliferation, inducing apoptosis and activating JNK signaling [253].

MO seed water extract reduces SIRT1 (sirtuin-1) and Bcl2 expression, promoting apoptosis in lymphoid and monocytoid cells, possibly through microRNAs that regulate cell proliferation [254]. MO extracts obtained using different solvents showed significant anticancer activity, with the n-hexane extract reducing HeLa cell viability by 50% at 416 μg/mL, supported by molecular docking analysis [255]. MO extract exhibited cytotoxic effects against HeLa and FaDu cells, both with an IC_50_ of 25 µg/mL, indicating its potential role in DNA fragmentation research [256].

The methanolic extract of MO leaves (MMLE) reduced cervical cancer cell growth, induced nuclear condensation, downregulated Jab1 (c-Jun activation domain-binding protein-1) expression, increased p27 expression, and arrested the cell cycle at G0/G1 phase. MMLE also increased ROS production and activated Caspase-3 in HeLa cells, suggesting its potential as a phytocompound targeting Jab1 in cervical cancer [257]. MO leaf fractions induced apoptosis via the mitochondrial pathway, arrested the cell cycle at the G2/M phase, suppressed telomerase activity, and inhibited HeLa cell colony formation [258].

MO seed extract improved insulin sensitivity in obese mice with triple-negative breast cancer (TNBC) tumors but worsened tumor progression when combined with chemotherapy. MO extracts decreased TNBC cell viability, induced apoptosis, and enhanced G2/M phase arrest [259]. MO compounds, including rutoside and vicenin-2, showed strong binding to BRCA-1 in breast cancer cell lines through molecular docking analysis [68]. Phytochemicals from MO demonstrated strong interactions with HIF-1α (hypoxia-inducible factor 1-alpha), VEGF, and GLUT1 receptors, showing favorable pharmacokinetics and potential as breast cancer inhibitors [260].

A nanosystem combining MO extract and caffeine in chitosan nanoparticles enhanced anticancer effects on MCF-7 cells by downregulating Her2 (Erb-B2 receptor tyrosine kinase 2), BRCA1, and BRCA2 while upregulating mTOR (mechanistic target of rapamycin kinase) expression [261]. MO leaf powder-based silver nanoparticles (MOLP-AgNPs) selectively induced apoptosis in MCF-7 cells via Caspase-3-dependent signaling and Akt inhibition [262]. MO aqueous leaf extract encapsulated in silver chitosan nanocomposites (AgCS-NCs) exhibited cytotoxicity against MCF-7 while preserving HEK-293 cell viability [263]. MO stem and leaf extracts triggered apoptosis in 4T1 breast cancer cells via Caspase-9 and -3 activation and an increased Bax/Bcl-2 ratio [264].

MO leaf extract protected against cyclophosphamide (CP)-induced ovarian damage in female Wistar albino rats. Pre-treatment with 250 mg/kg MO extract improved hormonal balance, reduced oxidative stress, and lowered inflammatory markers. It preserved ovarian and uterine structures, suggesting its potential as a pharmaceutical supplement for female reproductive health during chemotherapy [116].

Glucomoringin isothiocyanate from MO seeds inhibited PC-3 prostate cancer cell proliferation, inducing apoptosis via caspases, p53, Akt/MAPK, and Bax activation. GMG-ITC induced G2/M cell cycle arrest and increased early apoptosis proteins, suggesting its potential as a prostate cancer treatment [265]. MO and *Curcuma longa* extracts reduced PSA levels and p63 protein expression in BPH models, demonstrating an enhanced therapeutic effect when combined. MO extract also exhibited anti-inflammatory and anti-angiogenic effects by reducing IL-6, PCNA, and VEGF-A levels [266,267].

MO alkaloid extract (MOAE) inhibited A549 lung cancer cell proliferation and induced apoptosis through Caspase-3 and Caspase-9 activation. It caused S-phase arrest, suppressed cell migration, and inhibited the JAK2/STAT3 (Janus kinase 2/signal transducer and activator of transcription 3) signaling pathway, suggesting its role as a lung cancer therapeutic agent [23]. MO leaf polysaccharides (MOLP) reprogrammed tumor-associated macrophages, enhancing T-cell infiltration and immune response and thereby presenting a potential immunotherapy approach [268]. MO leaf extract (MLE) improved lung histology and antioxidant levels in a urethane-induced lung cancer model, demonstrating its potential as an alternative to toxic chemotherapy [269].

Moringa seeds and seed oil demonstrated anticancer properties against colon cancer. MO leaf extract combined with AgNPs was tested on azoxymethane (AOM)-induced colon cancer in rats, showing restoration of hematological and biochemical markers, reduction in tumor severity, and increased expression of TP53 and APC genes. This nano-extract prevented histopathological alterations and restored normal gene expression patterns [270].

MO extract alone and with AgNPs (61 nm) exhibited antioxidant and antibacterial activity, induced apoptosis, and increased p53 expression in HT-29 colon cancer cells. Both treatments arrested HT-29 cells at the G2/M phase and stimulated splenic cell growth [271]. MO-derived AgNPs reduced CTNNB1 and LRP6 gene expression while increasing LRP5, demonstrating a promising colon cancer treatment strategy [272].

MO peptides (MOPHs) showed significant antioxidant activity (FRAP: 1435 µmol TE/g) and inhibited Caco-2 colon cancer cell proliferation by up to 90.20%, with no cytotoxic effects on healthy colon cells [273]. Bio-fabricated AgNPs from MO leaf powder (MOLP-AgNPs) reduced HT-29 cell viability and expression of proliferation-related genes (Ki-67, Wnt, β-catenin, and cyclin D1) and metastasis markers (TGF-β and Snail) [274].

MO seed extract exhibited anticancer properties against Caco-2, MDA, and HepG-2 cancer cell lines, inducing apoptosis and cycle arrest by regulating tumor suppressor and anti-apoptotic proteins [275]. MO leaves demonstrated chemopreventative effects in colorectal cancer models by reducing tumor incidence and modulating enzyme activities [155,276]. Gold nanoparticles synthesized from MO have shown potential as antiproliferative agents in cancer therapies [277]. MO downregulated pro-inflammatory cytokines (IL-2, IL-6, and TNF-α), reducing inflammation-linked cancer progression [101,155,247]. However, MO combined with chemotherapy may worsen tumor progression in certain contexts, such as triple-negative breast cancer, necessitating careful use alongside conventional treatments [259].

A combination of MO, germinated brown rice, and *Cordyceps militaris* exhibited enhanced antioxidant and enzyme-inhibiting properties. The formulations promoted glucose consumption and anti-inflammatory activity without toxicity to normal and cancer cells [278]. MO fruit extract, rich in phenolics and flavonoids, induced apoptosis in HepG2 liver cancer cells through ROS production and Caspase-3 activation [279].

MO/ascorbic acid–selenium nanoparticles (MO/asc.-Se-NPs) significantly improved antioxidant and anti-inflammatory effects and liver enzyme levels in hepatocellular carcinoma (HCC)-induced Wistar rats, reducing IL-6 and alleviating liver tissue damage [280]. MO seed extracts exhibited antioxidative activity and reduced metabolic activity in HepG2 liver cancer cells, mitigating liver injury [281].

MO and green tea extracts reduced liver enzyme levels, enhanced antioxidant enzymes (CAT, GPx, and SOD), and downregulated oxidative stress markers in DOX-treated mice, showing hepatoprotective effects [282]. MO and *M. peregrina* leaf extracts exhibited cytotoxic and anti-migration effects on HepG2 cells, attributed to polyphenolic compounds [283].

MIC-1, a bioactive compound in MO Lam., selectively inhibited renal cell carcinoma (RCC) growth by targeting PTP1B-mediated Src/Ras/Raf/ERK signaling without affecting non-RCC cancer cell lines [284]. Moringin, derived from MO glucosinolate GMG, significantly reduced SH-SY5Y neuroblastoma cell growth by inducing apoptosis and inhibiting NF-κB nuclear translocation [285].

MO extracts from various plant parts exhibited significant anticancer effects on head and neck cancer (HNC) cells, with ethanolic extracts from stems showing the strongest apoptotic effects [286]. MO leaves demonstrated strong neuroprotective potential, with high radical scavenging and ROS reduction activities, suggesting therapeutic applications for neurodegenerative disorders [287].

MO methanol leaf extract (MOML) induced apoptosis in murine non-Hodgkin lymphoma (NHL) cells via mitochondrial dysfunction and MEK/ERK pathway inactivation [288]. Gold nanoparticles synthesized with MO leaf extract (MLE-AuNPs) showed cytotoxicity against Dalton’s lymphoma (DL) cells, causing cell cycle arrest and apoptosis through Bcl-2 downregulation and Bax, Cyt-c, and Caspase-3 upregulation [289].

Molecular docking identified hesperetin, gossypetin, and quercetin as promising anticancer compounds targeting EGFR and VEGFR-2, offering potential for improved NSCLC treatment [290]. Gold nanoparticles (AuNPs) synthesized from MO seeds exhibited antioxidant and antimicrobial activity and inhibited A549 lung cancer cell proliferation in a dose-dependent manner [291].

Silver nanoparticles (AgNPs) synthesized using plant extracts from MO, *Gymnema sylvestre*, and *Azadirachta indica* significantly downregulated VEGF and cyclin-D1, inhibiting angiogenesis and cell cycle progression in A549 lung cancer cells. AgNPs exhibited potent cytotoxic effects, surpassing the efficacy of plant extracts alone, highlighting their potential for lung cancer therapy with environmentally sustainable production [292].

**Figure 1:**
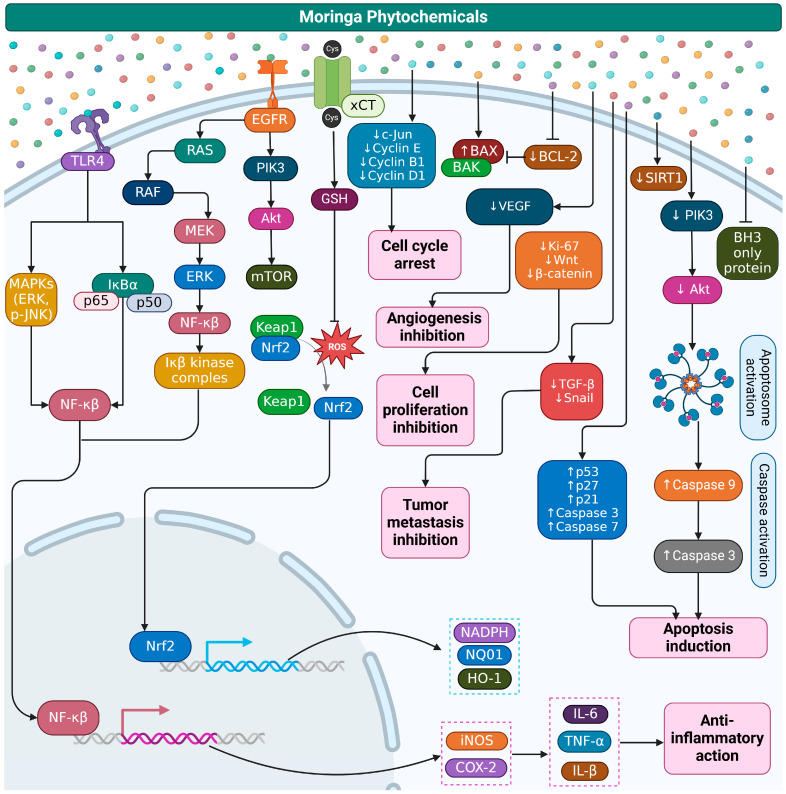
Mechanisms of Moringa’s Anti-Cancer Bioactivity. The anti-cancer potential of several MO extracts triggers multiple molecular pathways, modulating the expression of markers such as TNF-α; altering mitochondrial membrane potential; promoting ROS generation; downregulating COX-2, Wnt/β-catenin, NF-κB, and BCL-2 expression; and influencing antioxidant enzyme activities such as CAT, SOD, glutathione reductase, and GSH-Px. Additionally, the extracts enhance cell cycle arrest and upregulate markers such as BAX, 8-oxo-dG, apurinic sites, p53, GST (glutathione S-transferase), p-ERK1/2, AKT, and QR, contributing to their anticancer effects. Created in https://BioRender.com/c24l810, accessed on 28 February 2025.

A summary of the main pathway target by Moringa phytochemicals is shown in Figure 1. It includes the following: (i) Under oxidative conditions, the Keap1/Nrf2 complex in the cytoplasm dissociates into Keap1 and Nrf2, which are translocated into the nucleus. The transcription of detoxifying genes is induced only when the antioxidant response element (ARE) is bound to both mRNA and Nrf2. (ii) GSH is activated through intracellular secretion of cysteine, which is exported via the cysteine/glutamate antiporter (xCT). This process enhances the trapping of ROS. (iii) Concurrently, the epidermal growth factor (EGF) and EGFR interaction trigger the intracellular RAS-RAF-MEK-ERK pathway. This leads to the secretion of NF-kB subunits p50 and p65. Subsequently, the IkB/kinase complex stimulates the translocation of these subunits into the nucleus. Additionally, transcription of inducible nitric oxide synthase (iNOS) is initiated. (iv) Simultaneously, lipopolysaccharide (LPS)-induced activation of the Toll-like receptor-macrophage 2 (TLR4-MP2) promotes the transcription of COX-2, resulting in the nuclear translocation of the CREB-C/EBP complex. Moringa extracts show cytotoxic effects on cancer cells while promoting the proliferation of normal lymphocytes, indicating its dual role in immune modulation and cancer prevention [293].

The following table (Table 5) summarizes the in vitro and in vivo findings on Moringa’s potential to alleviate CDs.

## 5. Conclusions

*M. oleifera*, widely recognized as a “miracle tree”, offers a promising solution to combating chronic diseases due to its rich phytochemical composition and diverse pharmacological properties. Its applications span various domains, including nutrition, medicine, agriculture, and cosmetics, making it a versatile and invaluable natural resource.

The plant’s antioxidative, anti-inflammatory, and immunomodulatory properties significantly prevent and manage diseases such as diabetes, cardiovascular conditions, and obesity. Its potent wound-healing abilities further emphasize its therapeutic potential. Additionally, the plant’s high nutritional value, coupled with its affordability and accessibility, makes it a valuable tool for addressing malnutrition and improving public health, especially in resource-limited regions.

Despite its immense potential, challenges such as variability in bioactive compound concentrations, limited large-scale human studies, and issues with taste and acceptability limit its widespread application. Future research should prioritize standardizing extracts, formulation innovations, and large-scale clinical trials to unlock its full therapeutic potential. MO represents a sustainable and multi-functional solution to global health challenges, warranting further exploration and integration into clinical and non-clinical applications.

## 6. Future Directions

MO has shown potential in alleviating CDs due to its rich composition of bioactive compounds. Future directions for its use in this context should focus on expanding research and clinical applications. While preclinical studies suggest MO’s potential in treating various CDs, large-scale clinical trials are essential to validate its safety, efficacy, and dosage across diverse populations. Therefore, future research should prioritize the development of clinical trials to confirm MO’s effectiveness in treating CDs. Current studies are limited, and more robust trials could help establish standardized dosages and formulations. These trials should focus on CDs such as diabetes, cardiovascular conditions, obesity, cancer, and neurodegenerative diseases.

Standardized formulations of MO extracts must be developed to address variability in bioactive compound concentrations. This includes establishing quality control protocols to ensure consistency and reliability across therapeutic applications. Developing standardized Moringa-based products, such as teas or extracts, could also facilitate their use in clinical settings. These products should be easy to prepare and consume, particularly in regions where Moringa is readily available.

Despite MO’s great therapeutic potential, challenges related to taste acceptability remain. Investigating ways to improve the palatability of MO-based products could enhance their acceptance, particularly in functional foods or supplements. Further research into the molecular mechanisms of Moringa’s bioactive compounds could provide deeper insights into their therapeutic potential and aid in developing targeted treatments. Advanced techniques like computational modeling and high-throughput screening could help identify interactions with human biological pathways.

Expanding research into MO’s potential applications in emerging fields, such as microbiome modulation in CDs management, could further establish MO as a multipurpose resource. Addressing bioavailability, taste, and acceptability challenges would enhance its therapeutic applicability. Research into novel delivery systems, including encapsulation, nanoparticles, or combination therapies, could improve patient compliance and effectiveness.

Investigating the synergistic effects of MO with other natural products or pharmaceuticals holds promise for developing combination therapies, particularly for complex conditions like cancer. Additionally, establishing a clear regulatory framework for MO’s medicinal and nutraceutical use is crucial. Securing approvals from global regulatory bodies (e.g., FDA and EMA) would facilitate its widespread acceptance and integration into healthcare systems.

## Figures and Tables

**Table 1:** Taxonomical classification of *M. oleifera*.

Kingdom	Plantae
Sub-kingdom	Tracheobionta
Super division	Spermatophyta
Division	Magnoliophyta
Class	Magnoliopsida
Subclass	Dilleniidae
Order	Capparales
Family	Moringaceae
Genus	*Moringa*
Species	*oleifera*

**Table 2:** Proximal analysis of *Moringa oleifera* phytochemical composition.

Type of Compound	Extraction Process and Operational Conditions	Yield	Ref.
Phenols	MO leaf powder (50 g) was extracted using the ultrasonic extraction (UE) method with 70% ethanol at a 30:1 solvent-to-sample ratio, maintained at 50 °C for 40 min. The extract was then purified using organic solvent extraction followed by column chromatography.	962.6 mg RE/g	[14]
	MO leaf powder and DESs (choline chloride/citric acid) were mixed at a solid–liquid ratio of 1:20 g/mL. Ultrasonic extraction was performed at 420 W, with the temperature maintained at 50 °C. After 30 min, the extract was centrifuged at 5000 rpm for 10 min.	86.92 ± 1.34 mg GAE/g	[15]
Flavonoids	MO leaf powder was mixed with DESs (choline chloride/citric acid) at a solid–liquid ratio of 1:20 g/mL. Ultrasonic extraction was performed at 420 W, with the temperature maintained at 50 °C. After 30 min, the extract was centrifuged at 5000 rpm for 10 min.	49.73 ± 0.85 mg RT/g	[15]
Glucosinolates	MO leaves, seeds, and stems were extracted using 70% methanol and quantified by HPLC. MO leaves were also extracted using a solvent mixture of dimethyl sulfoxide, dimethyl formamide, and acetonitrile (1:1:1) and quantified by HPLC.	70 μmol/g leaves dw 80 μmol/g seeds dw <20 μmol/g stems dw	[16]
Isothiocyanates	MO leaves, immature seeds, mature seeds, pods, and petioles were extracted using 50% ethanol and quantified by HPLC.	7.52 ± 0.44 PIE/g leaves extract 13.9 ± 1.0 PIE/g immature seeds extract 27.84 ± 0.34 PIE/g mature seed extract 7.22 ± 0.80 PIE/g pods extract 17.9 ± 1.5 PIE/g petioles extract	[17]
Sterols	Cold pressing: MO oil was extracted from seeds using a cold-press system (2–6 L/h capacity) without applying heat. SOXE: MO oil was recovered from powdered seeds using petroleum ether at 60 °C for 6 h. Following extraction, a saponification process was performed, and both samples were analyzed using TLC and GC.	~8.25% (cold-pressing process) ~7.73% (SOXE process)	[18]
Fatty acids	Cold pressing: MO oil was extracted from seeds using a cold-press system (2–6 L/h capacity) without applying heat. SOXE: MO oil was recovered from powdered seeds using petroleum ether at 60 °C for 6 h. An esterification process was then performed, and both samples were analyzed using GC.	~10.4%	[18,19,20]
Alkaloids	MO leaf powder (10 g) was extracted with 50% ethanol for 24 h, followed by extraction with ethyl acetate and chloroform.	~30 g/10 Kg	[21,22,23]
Carotenoids	Maceration method: MO leaf powder (550 mg) was macerated in methanol for 6 h. The sample was then washed with 10 mL of saturated NaCl, followed by sequential washing with n-hexane, a 5% KOH/methanol mixture (for 3 h), and anhydrous Na_2_SO_4_. Finally, saponification and drying (24 h) were performed. SOXE: Oil was extracted from ground MO seeds (100 g) using the Soxhlet system with n-hexane (60–80 °C) as the solvent. SFE: A ground MO seed sample (100 g, 0.46 mm particle size) was loaded into the extraction vessel. Extraction was carried out at 40 °C and 403 bar for 30 min, with a total CO_2_ flow rate of 70 g/min. Ethanol was used as a co-solvent.	0.4798 mg/50 mg (maceration method) 15.20 ± 0.14 ppm (SOXE process) 16.63 ± 0.10 ppm (SFE process)	[24,25,26]
Vitamins	UAE was used for extraction. It was optimized for extracting vitamins using mixtures of ethanol and water (50:50).	~133.78 mg/100 g of fresh leaves ~11.50 mg/100 g dried leaves ~151.16 mg/100 g of seeds	[27,28]
Minerals	UAE was used for extraction. It was optimized for extracting minerals using mixtures of ethanol and water.	~135.32 mg/100 g of fresh leaves ~691.18 mg/100 g of dried leaves ~152.05 mg/100 g of seeds	[27,29,30]
Amino acids	UAE was used for extraction. It was optimized for extracting amino acids using mixtures of ethanol and water.	19 amino acids (~5.04 mg/100 g of dry matter)	[27,29,31]

DESs, deep eutectic solvents; GAE, gallic acid equivalents; GC, gas chromatography; HPLC, high-performance liquid chromatography; PIE, phenethyl isothiocyanate equivalent; RE, rutin equivalents; RT, rutin total; dw, dry weight; SFE, supercritical fluid extraction; SOXE, Soxhlet extraction; TLC, thin layer chromatography.

**Table 4:** Phytochemical extraction optimization.

Extraction Method	Solvent	Phytochemical	Concentration	Ref.
Ultrasound-assisted extraction—Deep eutectic solvents	Choline chloride/citric acid	TPC	86.92 ± 1.34 mg GAE/g	[15]
		TFC	49.73 ± 0.85 mg RT/g	
Pressurized liquid extraction	Water	TPC	24.10 mg GAE/g dw	[109]
		TFC	19.89 mg RE/g dw	
Ultrasound-assisted extraction	Ethanol (60%)	Quercetin	0.05708 mg/g	[110]
		Kaempferol	0.1345 mg/g	
UAE and microwave-assisted extraction	Ethanol (77%)	TPC	8.67 mg GAE/g DP	[111]
		TFC	69.47 mg CE/g DP	
Automated solvent extraction	Ethanol	TPC	34.201 mg GAE/g	[112]
		TFC	162.349 mg CE/g	

TPC: total phenolic content; TFC: total flavonoid content; GAE: gallic acid equivalents; dw: dry weight; RE: rutin equivalents; DP: dry plant; CE: catechin equivalents.

**Table 5:** In vitro and in vivo studies involving Moringa extracts to alleviate chronic diseases.

Source	Model	Extraction Type	Dose	Time	Results	Ref.
*M. oleifera* Lam.	Albino rats of both sexes	Methanolic (99.7%) extract	250 and 500 mg/kg	10 consecutive days	Dose-dependent anti-edematous effect of MO via downregulation of TNF-α and interleukin-1β.	[164]
*M. oleifera* (wild type)	Wistar rats by formaldehyde-induced arthritis model	Methanolic and aqueous extracts	150, 300, and 600 mg/kg dose	10 days	MO extracts reduced arthritis symptoms in rats in a dose-dependent manner.	[122]
*M. olifera* leaf	In vitro model of human glomerular epithelial cells (HGEC)	Methanolic extract	NA	NA	Methanolic extract of *Moringa oleifera* showed potential in reducing diabetic nephropathy-related inflammation and promoting kidney cell regeneration in vitro.	[294]
*M. oleifera*	Male Wistar rats	Methanolic extract	250 mg/kg b.wt	6 weeks	MO improved antioxidant levels and reduced inflammation in diabetic rats.	[159]
*M. oleifera* Lam.	Male C57BL/6J mice	MO seed extract	150 mg/kg orally administered	1 or 2 weeks	Moringa seed extract reduced inflammation and improved colonic health in ulcerative colitis models.	[295]
MO seed oil and lycopene	Male Sprague–Dawley rats	Seed oil extract	800 mg/kg b.wt and 20 mg/kg b.wt)	8 weeks	MO seed oil and lycopene reduced obesity markers in rats on a high-fat diet.	[166]
*M. peregrina*	Adult male Wister albino rats	Ethanolic extraction	300 mg/kg and 600 mg/kg orally administered	8 weeks	600 mg/kg MP leaf extract reduced obesity markers and improved liver health in high-fat diet-fed rats.	[168]
MO leaves	High-fat diet-induced obesity and cardiac damage in rats	Methanolic extract	200 mg/kg/bw and 400 mg/kg/bw orally administrated	12 weeks	400 mg/kg of MO improved heart health and antioxidant status in obese rats.	[173]
MO seed extract	Obese C57BL/6J male mice	NA	Standard low-fat diet or a very high-fat diet supplemented with MO seed extract	12 weeks	MO seed extract reduced body weight and improved glucose tolerance in mice.	[296]
MO leaves	Male Wistar rats	Ethanolic extract	300 mg/kg BW	14 weeks	MO extract reduced obesity and improved metabolic markers in high-fat diet-fed rats.	[174]
MO seed extract-containing MIC-1 with a curcuminoid-enriched turmeric extract (CTE)	RAW 264.7 murine macrophages	Heat water extract	MIC-1 (1, 5, or 10 μM) or CEM (curcumin concentration of 0.7, 3.5, or 7 μM)	8 h	MIC-1 demonstrated significant anti-inflammatory and antioxidant effects, reducing nitric oxide (NO) production at 1 μM and suppressing iNOS, IL-1β, and IL-6 gene expression at 5 μM; at 10 μM, MIC-1 effectively upregulated Nrf2 target genes (NQO1, GSTP1 (glutathione-S-transferase Pi 1), and HO1).	[297]
	Male Sprague–Dawley rats		MIC-1 at 250 or 500 mg/kg and CTE at 400 mg/kg	30 min before inducing inflammation	MSE showed a reduction in the carrageenan-induced rat paw edema (33% at 500 mg/kg MIC-1) comparable to aspirin (27% at 300 mg/kg), whereas CTE had no significant effect.	
GABA from MO leaves	RAW 264.7 cells model	GABA-enriched *Moringa oleifera* leaves fermented broth (MLFB)	31.25 µg/mL to 500 µg/mL	24 h	MLFB exhibited dose-dependent anti-inflammatory effects by inhibiting the secretion and intracellular expression of pro-inflammatory cytokines (IL-1β, IL-6, IL-8, and TNF-α); it also suppressed PGE2 and iNOS expression while downregulating TLR-4 and NF-κB mRNA levels, indicating its regulatory role in the NF-κB signaling pathway.	[162]
MO Lam. extract and MO-loaded nanoclay films	Albino rats	Methanolic extract	250 mg/kg and 500 mg/kg administered orally	48 h	MO Lam. extract and its nanoclay-based films show anti-inflammatory and anti-angiogenic properties, offering potential therapeutic applications for CDs.	[164]
MO seed oil	Swiss mice	MO seed oil is commercially available in Paraguay	Topical treatment using 1, 3, and 10 µL of MO seed oil	MO seed oil was applied topically	MO seed oil effectively reduces chronic skin inflammation and hyperproliferation, expanding its therapeutic potential for inflammatory skin diseases.	[163]
MO Lam. leaves	RAW 264.7 macrophages	MO proteins were extracted, precipitated with 90% (NH_4_)_2_SO, and dissolved in water	1–100 μg/mL	20 h	MO leaf peptides (1.33 mg/mL) exhibited potent antioxidant activity, inhibiting DPPH and ABTS radicals by 45.70% and 93.09%, respectively, with an ORAC value of 3.27 mM Trolox equivalent/g; at 100 μg/mL, they reduced nitric oxide production by 30.51%, demonstrating their anti-inflammatory potential.	[298]
Moringa seed	Caco-2, MDA, and HepG-2 cells	Methanolic extract	IC_50_ were 9.15 ± 1.18 µg/mL forCaCo-2, 4.85 ± 0.11 µg/mL for MDA, and 7.36 ± 0.22 µg/mL for HepG-2 cells	-	MO seed extract effectively induced cell cycle arrest at both G0/G1 and G2/M phases, inhibiting cell population growth; it promoted apoptosis by upregulating p53 and p21 expression (three- to sixfold) while downregulating Bcl-2 (threefold) in treated cells.	[275]
Moringa seeds	Female mice with diet-induced obesity bearing MDA-MB-231-derived xenograft tumors	Ethanolic extract	Moringa concentrate (MC) was added to a high-fat (D12492; 0.198% MIC-1; 0.6% MC)	-	Moringa supplementation improved metabolic health in mice with obesity and triple-negative breast cancer but may worsen tumor progression when combined with chemotherapy.	[259]
MO leaves	Male mice (CD-1)	Powder of freeze-dried fresh leaves	10% and 20% MO	12 weeks	Reduction in proinflammatory cytokines (MCP-1, IL-6, and TNF-α) and induction of differential expression genes (*IL-2*, *IL-6*, *TNF, IL-1ß*, and *INF-γ*).	[155]
MO leaves	Isoniazid-induced model	Suspensions of extract were prepared using sodium carboxymethyl cellulose (Na-CMC)	400 mg/kg BW	28 days	Oral suspensions of MO leaf extract reduced serum glutamate oxaloacetate transaminase and serum glutamate pyruvate transaminase levels, demonstrating potential as a novel hepatoprotective agent and an alternative to tablets for treating liver diseases.	[238]
MO leaves	Male Wistar rats	Aqueous extract	200 mg/kg	4 weeks	MO leaf extract significantly reduced lead-induced liver damage in male Wistar rats by reducing oxidative stress-induced DNA damage and maintaining hepatocyte integrity.	[237]
MO leaves	Male Wistar rats	MO leaves extracts loaded into niosomes	250 mg/kg	4 weeks	Pre- and co-treatment with MO leaf extract-loaded niosome nanoparticles can protect the heart against DOX-induced cardiotoxicity by suppressing oxidative stress, inflammation, and apoptosis.	[113]
Moringa leaves and seeds	Diabetic rats	Moringa leaf and seed extracts	4% Moringa	14 days	Co-administration of MO leaf/seed-supplemented diets and acarbose synergized managing diabetic cardiomyopathy, improving antioxidant status, and modulating biochemicals.	[196]
MO leaves	BV173 leukemic cell line and healthy peripheral blood mononuclear cells (PBMCs)	Ethanolic extract	IC_50_ = 125 ± 6 µg/mL for BV173 cell line;IC_50_ = 28 ± 3 µg/mL for PBMCs	48 h	MO leaf ethanol extract had immunostimulatory properties on normal lymphocytes and anti-tumor activity on leukemic cell lines, potentially benefiting patients with chronic myeloid leukemia.	[293]
MO leaves	Male Wistar rats	Methanolic extract	250 mg/kg orally administered	4 weeks	MO extract protected against lead acetate-induced kidney damage in rats due to its antioxidant, anti-inflammatory, and anti-apoptotic properties.	[247]
MO leaves	Human glomerular epithelial cells (HGEC)	Methanolic extract	-	-	MO showed potential in reducing diabetic nephropathy-related inflammation and promoting kidney cell regeneration in vitro.	[294]
MO seed oil	Male Wistar rats	Ethanolic extract	300 mg/kg	7 days	MO seed oil exhibited hepato-protective and nephroprotective properties, reducing liver and kidney damage caused by dichlorvos exposure in male Wistar rats.	[249]
MO seeds	Albino rats	Ethanolic extract	250 mg/kg/day	30 days	Niazimicin reduced malondialdehyde, cholinesterase, NO, and amyloid β by up to 59% and increased glutathione by 54%.	[229]
MO leaves	SH-SY5Y cell line	Ethanolic extract	IC_50_ = 2.7 ± 0.2 mg/mL	6 h	MO showed potential as a neuroprotective agent with 44% viable cells.	[287]
MO leaves	SH-SY5Y cell line	-	25, 50, and 100 μg/mL	-	MO leaf extract showed neuroprotective effects by suppressing oxidative stress and apoptosis in an Alzheimer’s disease model, potentially through Akt activation.	[299]
MO leaves	SH-SY5Y cell line	Methanolic extract	25 µg/mL	24 h	MO showed potential neuroprotective effects by reducing oxidative damage and promoting mitochondrial regulation, providing essential nutrients for a healthy diet; MO improved cell viability and reduced oxidative damage to 25 µg/mL.	[150]
MO leaves peptides	Spontaneously hypertensive rats	MO leaf protein was hydrolysated and ultra-filtrated	ACE IC_50_ = 0.29 ± 0.13 mM for LGF and IC_50_ = 0.31 ± 0.04 mM for GLFF; Renin IC_50_ = 1.88 ± 0.08 mM for LGF and IC_50_ = 2.80 ± 0.08 mM for GLFF	-	MO leaf peptides (LGF and GLFF) reduced systolic blood pressure by 19.4 mmHg and diastolic by 12 mmHg in rats.	[199]
Moringa seeds	Wistar strain albino rats	NA	Dietary inclusions of Moringa seed (5% and 10%)	2 weeks	Moringa seed acted as an ACE inhibitor and modulates the antioxidant system in hypertensive rats but did not affect ACE gene expression.	[300]
MO seeds	Male New Zealand Dutch strain albino rabbit	Methanolic extract	2 mg/wound	14 days	MO seed extract accelerated wound closure rates, increasing the expression of *TGF-β1*, VEGF, and type I collagen and decreasing the inflammatory markers and relative gene expression of *IL-1β* and *TNF-α*.	[183]
MO leaves	Diabetic rats with methicillin-resistant *S. aureus-* or *P. aeruginosa*-infected wounds	Methanolic extraction	MO (10%*w*/*w* and 20% *w*/*w)* formulated using soft paraffin, liquid paraffin, and emulsifying wax	10% *w*/*w* or 20% *w*/*w* of MO extract formulation once daily for 20 days	MO leaf extract promoted the healing of infected wounds in diabetic rats with MRSA infections but was less effective in those with *P. aeruginosa* infections.	[186]
	HaCaT cell line		1–1000 µg/mL	24 h	MO extract increased the expression of *VEGF* and *TGF-β1* genes.	
MO seed oil	Healthy, immunosuppressed, and diabetic female Swiss mice	NA	Pure oil (20 µL)	20 days	MO seed oil may accelerate chronic skin wound healing by improving collagen content and the number of myofibroblasts, with oleic acid partially responsible for this effect.	[187]

NA: not applicable.

## Data Availability

Not applicable.
